# Ethanolamine Signaling Promotes *Salmonella* Niche Recognition and Adaptation during Infection

**DOI:** 10.1371/journal.ppat.1005278

**Published:** 2015-11-13

**Authors:** Christopher J. Anderson, David E. Clark, Mazhar Adli, Melissa M. Kendall

**Affiliations:** 1 Department of Microbiology, Immunology, and Cancer Biology, University of Virginia School of Medicine, Charlottesville, Virginia, United States of America; 2 Department of Biochemistry and Molecular Genetics, University of Virginia School of Medicine, Charlottesville, Virginia, United States of America; University of California Davis School of Medicine, UNITED STATES

## Abstract

Chemical and nutrient signaling are fundamental for all cellular processes, including interactions between the mammalian host and the microbiota, which have a significant impact on health and disease. Ethanolamine is an essential component of cell membranes and has profound signaling activity within mammalian cells by modulating inflammatory responses and intestinal physiology. Here, we describe a virulence-regulating pathway in which the foodborne pathogen *Salmonella enterica* serovar Typhimurium (*S*. Typhimurium) exploits ethanolamine signaling to recognize and adapt to distinct niches within the host. The bacterial transcription factor EutR promotes ethanolamine metabolism in the intestine, which enables *S*. Typhimurium to establish infection. Subsequently, EutR directly activates expression of the *Salmonella* pathogenicity island 2 in the intramacrophage environment, and thus augments intramacrophage survival. Moreover, EutR is critical for robust dissemination during mammalian infection. Our findings reveal that *S*. Typhimurium co-opts ethanolamine as a signal to coordinate metabolism and then virulence. Because the ability to sense ethanolamine is a conserved trait among pathogenic and commensal bacteria, our work indicates that ethanolamine signaling may be a key step in the localized adaptation of bacteria within their mammalian hosts.

## Author Summary

Chemical signaling underlies all cellular processes. Bacteria rely on chemical signaling to gain information about the local environment and precisely regulate gene expression. Ethanolamine is an abundant molecule within mammalian hosts that plays an important role in mammalian physiology and also serves as a carbon and nitrogen source for bacteria. Here we show that the foodborne pathogen *Salmonella enterica* exploits ethanolamine as a signal of distinct host environments to coordinate metabolism and virulence, which enhances disease progression during infection. The ability to sense ethanolamine is conserved in diverse bacteria; thus, these studies reveal that ethanolamine signaling may be important for bacterial adaptation to the mammalian host.

## Introduction

Chemical and nutrient signaling mediate diverse biological processes, and underlie interactions among the mammalian host, the resident microbiota, and invading pathogens [1]. Ethanolamine is abundant in cell membranes, as a component of phosphatidylethanolamine as well as in modified lipid molecules such as *N*-acylethanolamines [2]. These ethanolamine-containing compounds play important roles in mammalian cell signaling and influence diverse physiological effects, including cytokinesis, immunomodulation, food intake and energy balance [2–4]. Ethanolamine is abundant in the intestinal tract due to the turnover and exfoliation of enterocytes and bacterial cells [5,6], and intracellular pools of ethanolamine are maintained by low and high affinity uptake systems as well as through internal recycling of phosphatidylethanolamine [7–10].

Bacterial pathogens compete for nutrients with the resident microbiota and rely on environmental cues to control virulence gene expression. *Salmonella enterica* serovar Typhimurium (*S*. Typhimurium) is a facultative intracellular pathogen and a leading cause of acute gastroenteritis, which can progress to systemic infection in susceptible individuals [11]. *S*. Typhimurium encodes two type three secretion systems (T3SSs) that are important for pathogenesis. *S*. Typhimurium uses the T3SS encoded within the *Salmonella* pathogenicity island (SPI)-1 to invade intestinal epithelial cells and penetrate to the lamina propria [12]. There, *S*. Typhimurium is taken up by macrophages, where it survives and replicates. Intracellular survival is mediated by the T3SS and effectors encoded in SPI-2 [13–15]. Ethanolamine can serve as a carbon and/or nitrogen source for bacteria in the intestine as well as within epithelial cells [16,17]. The aim of this work was to determine whether *S*. Typhimurium relies on ethanolamine as a signal to coordinate gene expression and augment virulence *in vivo*. Here, we show that the intramacrophage environment promotes expression of the ethanolamine utilization transcription factor EutR, which directly activates SPI-2. Moreover, we demonstrate that EutR signaling during systemic infection is specific to the intracellular environment and is important for robust *S*. Typhimurium dissemination. Altogether, our findings suggest that ethanolamine, an intrinsic component of bacterial and mammalian cell membranes, functions as a signal to modulate metabolism and virulence and suggest a new layer of complexity in chemical signaling that underlies pathogenicity.

## Results and Discussion

### EutR contributes to dissemination *in vivo*

**Fig 1:**
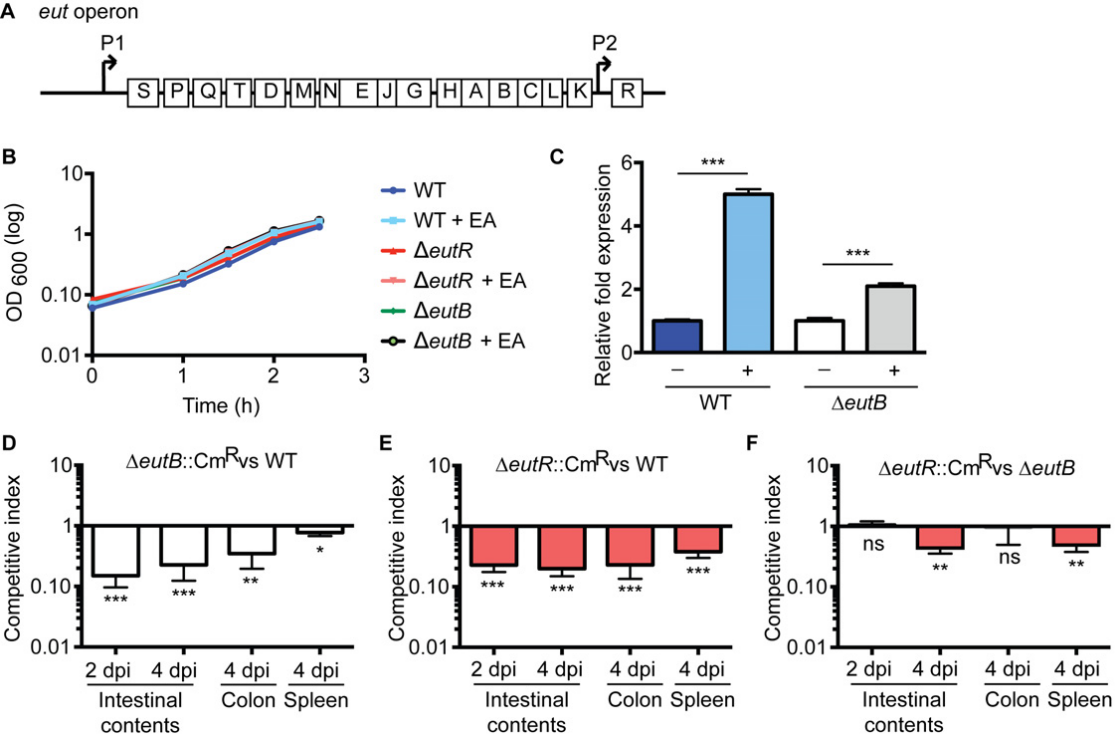
EutR in pathogen-microbiota-host interactions. (**A**) Schematic of the *eut* operon. (**B**) *In vitro* growth curve of S. Typhimurium WT (SL1344), Δ*eutR* (CJA009), or Δ*eutB* (CJA020) strains in LB without or with supplementation of 5 mM ethanolamine (EA). Each data point shows the average of three independent experiments. (**C**) qRT-PCR of *eutR* in WT or the Δ*eutB* (CJA020) *S*. Typhimurium strains grown in Dulbecco’s Modified Eagle Medium (DMEM) or DMEM supplemented with 5 mM EA. n = 3; error bars represent the geometric mean ± standard deviation (SD); *strB* was used as the endogenous control. (**D-F**) Competition assays between (**D**) Δ*eutB*::Cm^R^ (CJA018) and WT strains; (**E**) Δ*eutR*::Cm^R^ (CJA007) and WT strains; or (**F**) Δ*eutR*::Cm^R^ (CJA007) and Δ*eutB* (CJA020) strains. Mice were orogastrically inoculated with 1:1 mixtures of indicated strains. Colony forming units (cfu) were determined at indicated time points. Each bar represents a competition index (CI). Horizontal lines represent the geometric mean value ± standard error (SE) for each group (n = 2 litters (6–8 animals)). *, *P* ≤0.05; **, *P* ≤ 0.005; ***, *P* ≤0.0005; *P* > 0.05 = ns.

Genes encoding for ethanolamine metabolism are clustered in the *eut* operon [18] (Fig 1A). In the *Enterobacteriaceae*, expression of this operon is regulated by the *eut*-encoded transcription factor EutR. EutR is constitutively expressed at low levels from its own promoter and binds to the promoter region immediately upstream of *eutS*. In the presence of ethanolamine and vitamin B_12_, EutR activates transcription of this operon [19,20]. In enterohemorrhagic *Escherichia coli* (EHEC), EutR senses ethanolamine to activate virulence gene expression *in vitro*, independently of ethanolamine metabolism [19,21,22]. To determine whether EutR influences *S*. Typhimurium disease progression during infection, we generated an *eutR* deletion strain (Δ*eutR*) that cannot sense ethanolamine as well as an *eutB* deletion strain (Δ*eutB*) that lacks the large subunit of the ethanolamine ammonia lyase, and thus is unable to catabolize ethanolamine. The *eutR* and *eutB* mutations did not result in a general loss of fitness, as the Δ*eutR* and Δ*eutB* strains exhibited no measurable growth defects *in vitro* (Fig 1B). Importantly, the *eutB* mutation is nonpolar as this mutant can respond to ethanolamine (Fig 1C). Subsequently, we performed competitive infections in which streptomycin-treated mice were orally infected with an equal mixture of wild type (WT) and Δ*eutB* (Δ*eutB*::Cm^R^) strains or the WT and Δ*eutR* (Δ*eutR*::Cm^R^) strains. *S*. Typhimurium infection presents as intestinal outgrowth, invasion of epithelial cells, and subsequent uptake by macrophages and dissemination to secondary lymphoid tissue. Therefore, to monitor the course of *S*. Typhimurium infection, we analyzed the number of recovered bacteria from the intestinal contents, the colon, and the spleen. At 2 and 4 days post infection (dpi), the Δ*eutR* and Δ*eutB* strains were significantly outcompeted by the WT strain in intestinal contents (Fig 1D and 1E). These data underscore the importance of ethanolamine metabolism in *S*. Typhimurium colonization of the intestinal tract, and these findings are consistent with previous work by Thiennimitr *et al*., who showed that ethanolamine metabolism provides a growth advantage to *S*. Typhimurium during intestinal colonization [17].

Defects in ethanolamine metabolism have been reported to result in mild or no attenuation during *S*. Typhimurium systemic infection [23,24]; however the role of EutR, specifically, in contributing to dissemination has not been investigated. Therefore, to assess this, we harvested the colons and the spleens of infected mice at 4 dpi. The Δ*eutR* and Δ*eutB* strains were both recovered at significantly lower numbers than WT from the colon and spleen; however, the competition indices measured from the spleen from the Δ*eutR*/WT infections were significantly greater than between WT and the Δ*eutB* strain (*P* = 0.002). These findings led us to hypothesize that EutR plays a more extensive role in *S*. Typhimurium pathogenesis that is distinct from its function to promote ethanolamine metabolism.

To test this, we performed competition infections between the Δ*eutB* and Δ*eutR* (Δ*eutR*::Cm^R^) strains. At 2 days post infection (dpi), the Δ*eutR* and Δ*eutB* strains were recovered at similar numbers from intestinal contents (Fig 1F), indicating that at this initial stage of colonization, EutR functions to drive ethanolamine metabolism. However, at 4 dpi, which is a time point consistent with the progression to systemic infection [25], the Δ*eutR* strain was significantly outcompeted by the Δ*eutB* strain (Fig 1F). Significantly, although equal numbers of the Δ*eutR* and Δ*eutB* strains were recovered from the colon, the Δ*eutR* strain was significantly outcompeted by the Δ*eutB* strain in the spleen (Fig 1F). These data suggest that EutR, independent of its role in ethanolamine metabolism, is important to *S*. Typhimurium dissemination during infection.

### EutR does not influence invasion of epithelial cells

**Fig 2:**
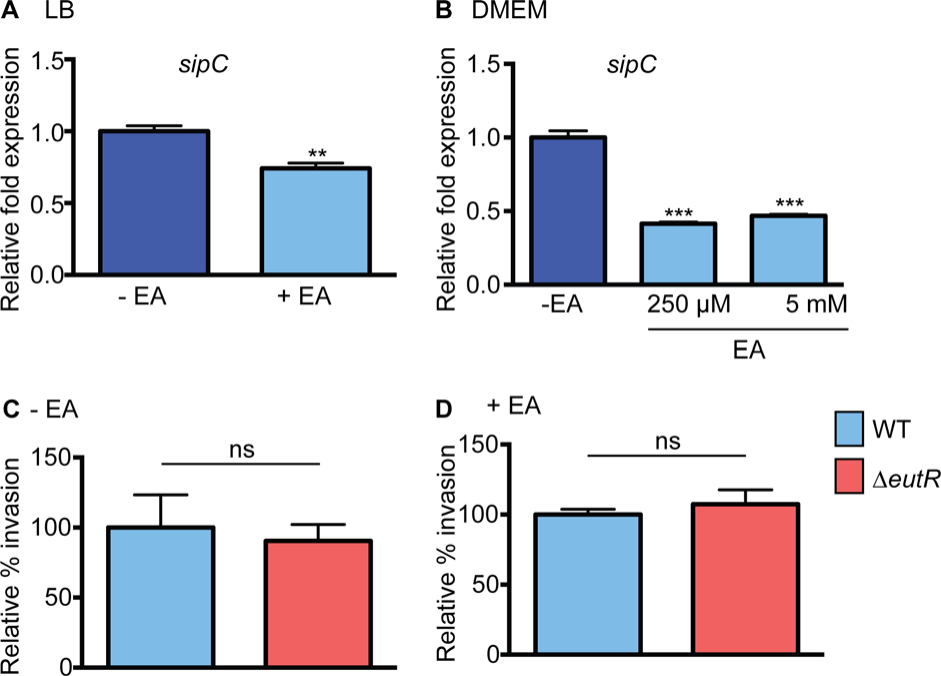
Effect of ethanolamine and EutR on SPI-1. (**A**) qRT-PCR of *sipC* from WT *S*. Typhimurium (SL1344) grown in LB or LB supplemented with 5 mM ethanolamine (EA). (**B**) qRT-PCR of *sipC* from WT *S*. Typhimurium (SL1344) grown in DMEM or DMEM supplemented with ethanolamine (EA) as indicated. For (**A**) and (**B**), n = 3; error bars represent the geometric mean ± SD. Statistical significance is shown relative to cells grown without EA supplementation; *strB* was used as the endogenous control. (**C**) Invasion of HeLa cells by WT (SL1344) and the Δ*eutR* (CJA009) strains. Mean ± SE of nine independent experiments. (**D**) Invasion of HeLa cells by WT (SL1344) and the Δ*eutR* (CJA009) strains. Mean ± SE of six independent experiments with supplementation of 5 mM EA. **, *P* ≤ 0.005; *P* > 0.05 = ns.

To further explore how ethanolamine signaling contributes to *S*. Typhimurium dissemination, we examined *S*. Typhimurium virulence gene expression *in vitro*. To examine ethanolamine-mediated expression of SPI-1, we measured expression of *sipC*, a SPI-1 encoded translocase that plays a role in invasion of epithelial cells [26]. For this, we grew *S*. Typhimurium in LB, which induces SPI-1 expression [27] as well as in DMEM used for cell culture assays. In both cases, expression of *sipC* was slightly decreased when ethanolamine was included in the culture medium (Fig 2A and 2B and S1 Fig). To determine whether the ethanolamine-dependent decrease in *sipC* expression impacted *S*. Typhimurium invasion of epithelial cells, we infected HeLa cells with either WT *S*. Typhimurium or the Δ*eutR* strain. WT *S*. Typhimurium and Δ*eutR* invaded HeLa cells at nearly equivalent levels in the presence or absence of ethanolamine (Fig 2C and 2D). Because we did not observe EutR-dependent effects on epithelial invasion under the specified conditions, the influence of ethanolamine at this stage in *S*. Typhimurium dissemination was not pursued further.

### Ethanolamine influences SPI-2 expression

**Fig 3:**
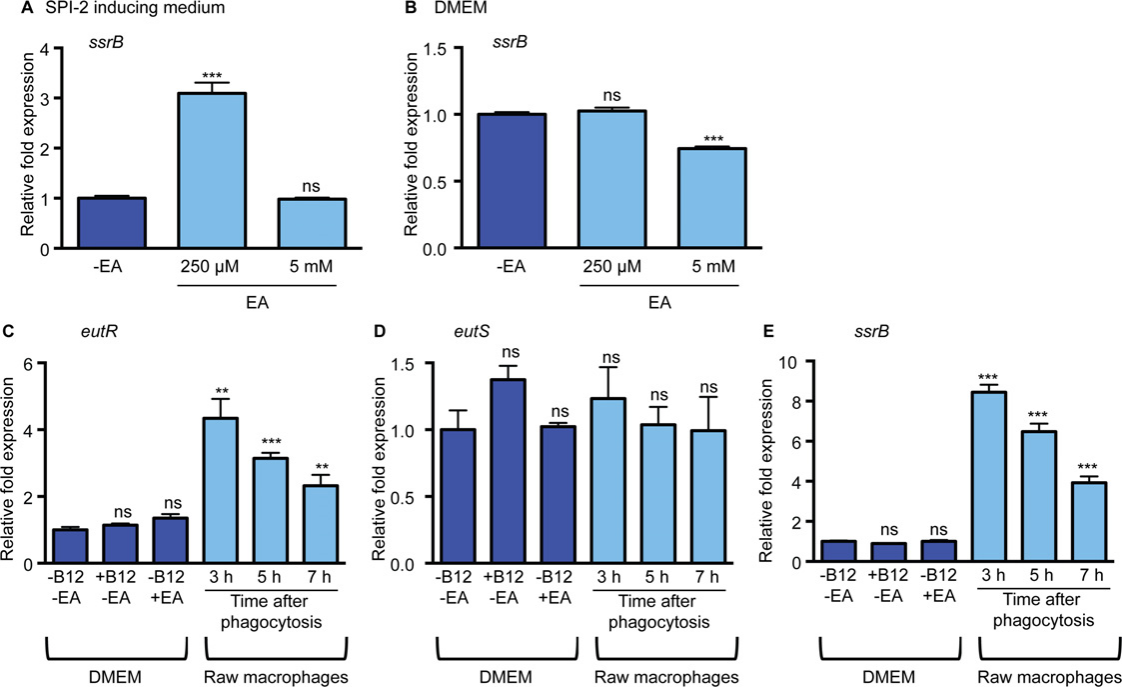
The impact of ethanolamine on SPI-2 expression *in vitro*. (**A**) qRT-PCR of *ssrB* from RNA isolated from the *S*. Typhimurium (SL1344) grown in SPI-2 inducing medium with ethanolamine (EA) supplementation as indicated. Statistical significance is shown relative to cells grown without EA supplementation. (**B**) qRT-PCR of *ssrB* from RNA isolated from the *S*. Typhimurium (SL1344) grown in DMEM with EA supplementation as indicated. Statistical significance is shown relative to cells grown without EA supplementation. (**C**) qRT-PCR of *eutR* from RNA isolated from *S*. Typhimurium (AJK61) grown in DMEM with supplementation as indicated or after phagocytosis in RAW macrophages. Statistical significance relative to cells grown in DMEM is indicated. (**D**) qRT-PCR of *eutS* from RNA isolated from *S*. Typhimurium (AJK61) grown in DMEM with supplementation as indicated or after phagocytosis in RAW macrophages. Statistical significance relative to cells grown in DMEM is indicated. (**E**) qRT-PCR of *ssrB* from RNA isolated from the *S*. Typhimurium strain (AJK61) grown in DMEM with supplementation as indicated or after phagocytosis in RAW macrophages. Statistical significance relative to cells grown in DMEM is indicated. For all, n = 3; error bars represent the geometric mean ± SD; *strB* was used as the endogenous control. *, *P* ≤ 0.05; **, *P* ≤ 0.005; ***, *P* ≤0.0005; *P* > 0.05 = ns.

Next, we investigated whether ethanolamine impacted SPI-2 expression. SsrB is a SPI-2 encoded transcriptional regulator that is required for expression of all the SPI-2-encoded genes, as well as for expression of effectors and virulence genes encoded outside of SPI-2 [28–31]. To test the influence of ethanolamine, we measured expression of *ssrB* in low magnesium, minimal medium, a condition that induces SPI-2 expression [32] (S2 Fig) without supplementation or with supplementation of 250 μM or 5 mM ethanolamine. These concentrations were used because 250 μM ethanolamine was the lowest concentration with which we could readily detect EutR expression (S3 Fig), whereas 5 mM is similar to ethanolamine concentrations in the gastrointestinal tract [33]. When 250 μM ethanolamine was added to the SPI-2 inducing medium, expression of *ssrB* was significantly increased compared to medium without supplementation, but unchanged when 5 mM ethanolamine was added (Fig 3A). These data suggest that ethanolamine may enhance the response of *S*. Typhimurium in adapting to the intramacrophage environment. Expression of SPI-2 is tightly regulated and is induced specifically in the intracellular environment [34], or in conditions that mimic the intracellular environment. In accordance, *ssrB* expression was not induced in DMEM or LB when ethanolamine was supplemented to the medium (Fig 3B and S4 Fig), indicating that ethanolamine in and of itself does not override additional regulatory factors that direct *ssrB* expression. However, *ssrB* expression was decreased in DMEM with the addition of 5 mM ethanolamine (Fig 3B). Altogether these data raised the possibility that ethanolamine signaling enhances niche recognition.

Macrophages play a significant role in the pathogenesis of *S*. Typhimurium infection by providing protected sites for intracellular replication and a means of dissemination [35]. Robust expression of EutR requires ethanolamine as well as the cofactor vitamin B_12_ [20]. *S*. Typhimurium synthesizes vitamin B_12_ under anaerobic conditions [36]; however, *S*. Typhimurium must acquire ethanolamine from the environment [18]. Therefore, we investigated whether the intracellular environment induces *eutR* expression. For this, we infected macrophages with *S*. Typhimurium in the absence of any exogenous ethanolamine or vitamin B_12_. Subsequently, RNA was extracted from internalized *S*. Typhimurium at 3, 5, and 7 h post phagocytosis, and *eutR* transcript levels were analyzed and compared to *eutR* transcript levels from *S*. Typhimurium grown in the absence of macrophages. Expression of *eutR* was significantly increased in phagocytized *S*. Typhimurium throughout infection compared to cells grown in the absence of macrophages (Fig 3C and S5 Fig). Moreover, neither vitamin B_12_ or ethanolamine alone activated *eutR* expression in tissue culture medium, indicating that the intramacrophage environment is conducive to EutR-dependent signaling.

The addition of ethanolamine and vitamin B_12_ to SPI-2 inducing medium or DMEM resulted in an increase in expression of the *eut* operon (as indicated by *eutS* expression (Fig 1A)) that corresponded with an increase in *eutR* expression (S6 Fig). Notably, the *eut* operon was not induced within macrophages (Fig 3D). These findings indicate that the ethanolamine metabolic genes are not activated within the intramacrophage environment. Interestingly, the expression pattern of *eutR* in internalized *S*. Typhimurium was similar to *ssrB* expression (Fig 3E); therefore, we hypothesized that EutR regulates SPI-2 expression.

### EutR directly regulates SPI-2 expression

**Fig 4:**
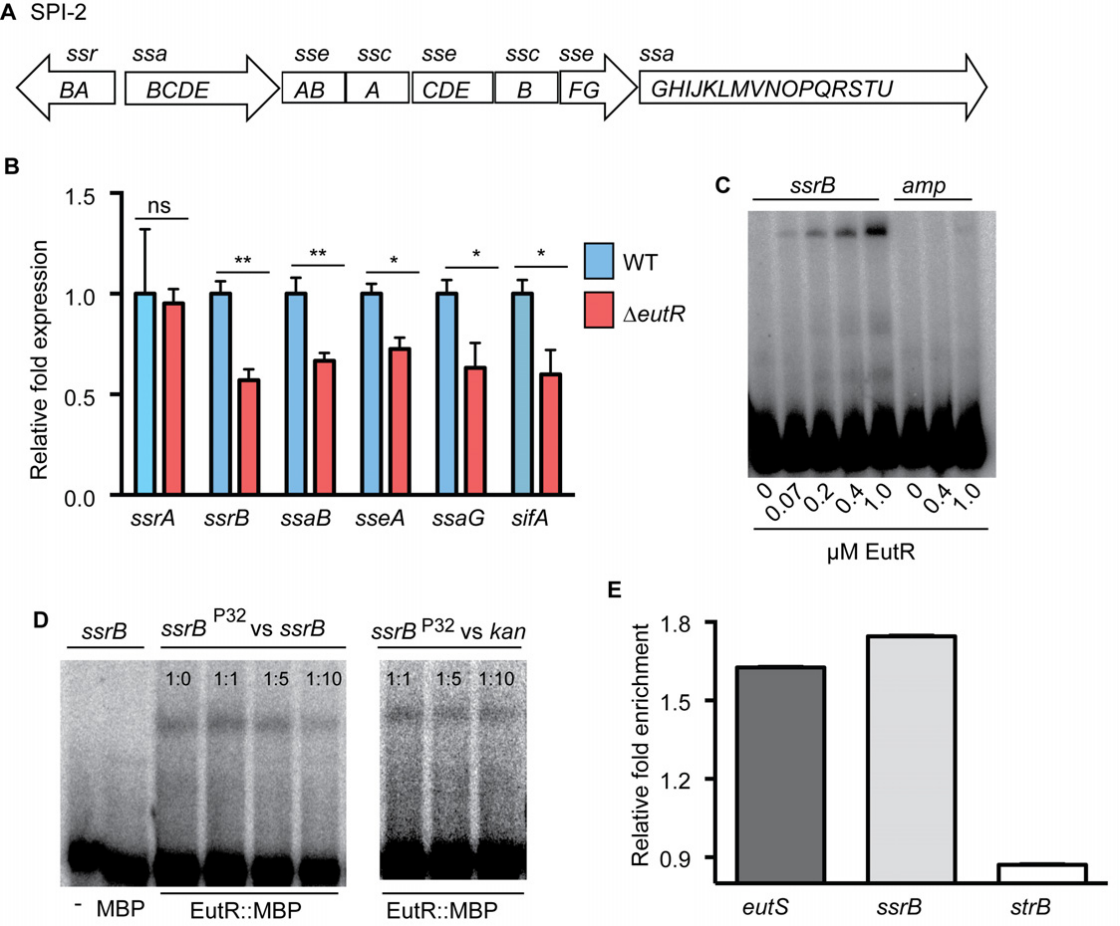
EutR regulates SPI-2 expression. (**A**), Schematic of SPI-2. (**B**) qRT-PCR analysis of SPI-2-encoded and associated (*sifA*) genes from RNA isolated from *S*. Typhimurium (AJK61) or the Δ*eutR* (CJA023) strains after 5 h phagocytosis in RAW macrophages. n = 3; error bars represent the geometric mean ± SD; *strB* was used as the endogenous control. (**C**) EMSAs of *ssrB* and *amp* (ampicillin) with EutR::MBP. (**D**) EMSAs of *ssrB* with MBP or EutR::MBP. Also, competition EMSAs with EutR::MBP. The assay was performed with increasing amounts of unlabeled *ssrB* promoter probe. A competition assay was also performed using the *kan* promoter as a negative control. The ratios represent hot:cold probe. (**E**) qPCR showing enrichment of *eutS*, *ssrB*, and *strB* from *in vivo* ChIP of EutR::MBP (n = 2). *, *P* ≤ 0.05; **, *P* ≤ 0.005; ***, *P* ≤0.0005; *P* > 0.05 = ns.

SPI-2 contains four major operons that encode a T3SS, chaperone and effector proteins, as well as the transcriptional regulator SsrB (Fig 4A). To test our hypothesis, we examined transcription of *ssrB* and one gene from each of the other major operons encoded in SPI-2 using RNA harvested from phagocytized WT or Δ*eutR S*. Typhimurium strains. Transcription of *ssrB* was significantly decreased in the Δ*eutR* strain compared to WT (Fig 4B and S7 Fig), and we measured a concomitant decrease in expression of all the SPI-2 operons, as well as the SPI-2-associated effector *sifA* (Fig 4B and S8 Fig). Expression of SPI-2 encoded and associated factors enhances the intrinsic ability of *S*. Typhimurium to withstand and disrupt host defense mechanisms [37,38], and these data revealed that EutR influences this critical aspect of *S*. Typhimurium virulence.

SsrB is a response regulator that comprises a two component system with the sensor kinase SsrA (also referred to as SpiR) [13,15]. SsrA autophosphorylates in response to the acidic environment of the *Salmonella*-containing vacuole (SCV) within host cells [39,40], which initiates a signaling cascade that promotes SsrB activity as well as *ssrAB* expression [41]. Importantly, the *ssrB* gene contains its own promoter [41]. The genetic data indicated that EutR influenced expression of *ssrB* and downstream targets, but that EutR did not impact *ssrA* expression (Fig 4B and S8 Fig), indicating that EutR may regulate SPI-2 expression by binding the *ssrB* promoter. To examine this, we purified an EutR::MBP fusion protein. Electrophoretic mobility shift assays (EMSAs) indicated that EutR directly binds the *ssrB* promoter to activate expression of SPI-2 (Fig 4C). To confirm specificity of binding, EMSAs with purified MBP alone as well as competitions assays with unlabeled probes were performed. MBP alone did not bind the *ssrB* promoter (Fig 4D). Furthermore, EutR binding was outcompeted by the addition of unlabeled *ssrB* probe; however, the addition of unlabeled *kan* probe, as a negative control reaction, showed no competition (Fig 4D).

Consistent with these results, there was a significant enrichment of the *ssrB* promoter when EutR-DNA interactions were analyzed using *in vivo* chromatin immunoprecipitation followed by qPCR (Fig 4E). As a positive control, we also measured enrichment of the *eutS* promoter, an established binding target of EutR [19], and observed similar enrichment of both targets (Fig 4E); *strB* DNA was used as a negative control. Control of *ssrB* expression is complex and also includes activation by additional TCS PhoP/PhoQ and EnvZ/OmpR, which respond to signals within the *Salmonella* containing vesicle (SCV) [41,42]. Our findings suggest that EutR-dependent activation of *ssrB* enables *S*. Typhimurium to integrate intrinsic information regarding the host cell through ethanolamine signaling with SCV-specific signals to coordinate efficient spatiotemporal expression of SPI-2 and SPI-2 associated effectors.

### EutR enhances intramacrophage survival

**Fig 5:**
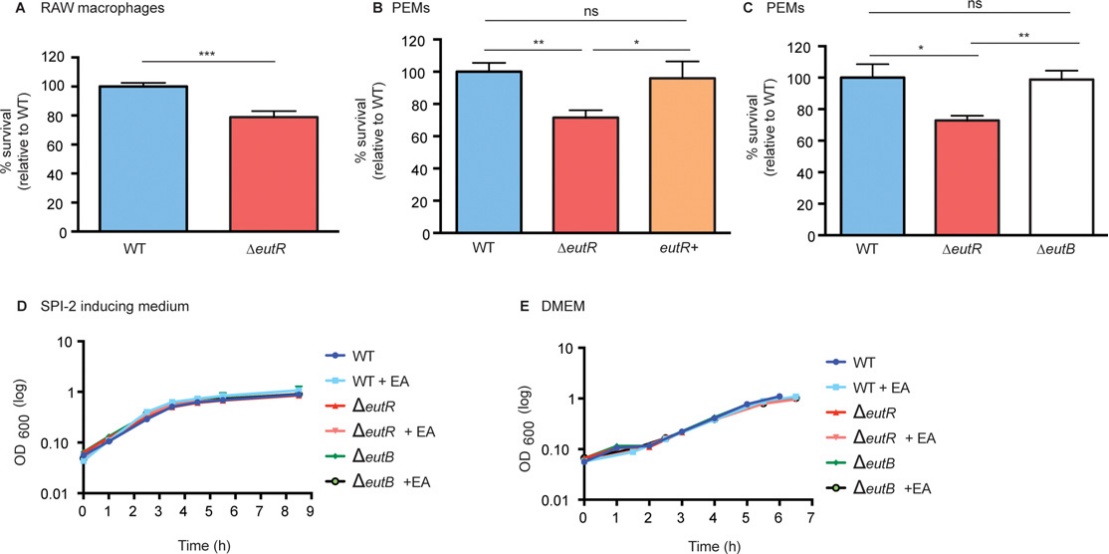
EutR enhances S. Typhimurium survival within macrophages. (**A**) Intramacrophage survival and replication of *S*. Typhimurium (AJK61) and the Δ*eutR* (CJA023) strains after 5 h phagocytosis in RAW macrophages (error bars represent the geometric mean value ± SE of 24 independent experiments). (**B**) Intramacrophage survival and replication of *S*. Typhimurium (CJA034), Δ*eutR* (CJA032), and Δ*eutR* complemented with *eutR* (*eutR*+) (CJA033) strains after 5 h phagocytosis in peritoneal exudate macrophages (PEMs) (error bars represent the geometric mean value ± SE of nine independent experiments). (**C**) Intramacrophage survival and replication of *S*. Typhimurium (AJK61), Δ*eutR* (CJA023) and Δ*eutB* (CJA028) strains after 5 h phagocytosis in PEMs (error bars represent the geometric mean value ± SE of six independent experiments). (**D**) *In vitro* growth curve of S. Typhimurium WT (SL1344), Δ*eutR* (CJA009), or Δ*eutB* (CJA020) strains in SPI-2 inducing medium without or with supplementation of 5 mM ethanolamine (EA). Each data point shows the average of three independent experiments. (**E**) *In vitro* growth curve of S. Typhimurium WT (SL1344), Δ*eutR* (CJA009), or Δ*eutB* (CJA020) strains in tissue culture medium without or with supplementation of 5 mM ethanolamine (EA). Each data point shows the average of three independent experiments. *, *P* ≤ 0.05; **, *P* ≤ 0.005; ***, *P* ≤ 0.0005; *P* > 0.05 = ns.

Next, we tested the consequences of EutR-dependent activation of SPI-2 on *S*. Typhimurium fitness during macrophage infection. Following infection of RAW or peritoneal exudate macrophages (PEMs), the Δ*eutR* strain was recovered at significantly lower numbers compared to the WT strain (Fig 5A–5C and S9 Fig). Additionally, complementation of the *ΔeutR* strain with *eutR* expressed from the native promoter (*eutR*+) restored intracellular survival to WT levels during primary macrophage infection (Fig 5B).

To verify that the defect in the Δ*eutR* strain was not the result of a defect in ethanolamine metabolism, we assessed survival of the Δ*eutB* strain within PEMs. The Δ*eutB* strain was recovered at similar numbers to WT and at significantly higher numbers than the Δ*eutR* strain (Fig 5C). Importantly, the Δ*eutR* mutant grows similarly to WT and Δ*eutB* strains in SPI-2 inducing medium and tissue culture medium with or without the addition of ethanolamine (Fig 5D and 5E), confirming that the decrease in intracellular survival is not a result of a EutR-dependent growth defect. These findings indicate that ethanolamine-associated signaling, but not catabolism, impacts *S*. Typhimurium survival within macrophages. Moreover, these findings, in conjunction with lack of *eut* operon induction within macrophages (Fig 3D), reveals that *S*. Typhimurium relies on EutR to direct gene expression in a manner that is particular to a specific niche.

### EutR provides a gauge of the intracellular environment *in vivo*

**Fig 6:**
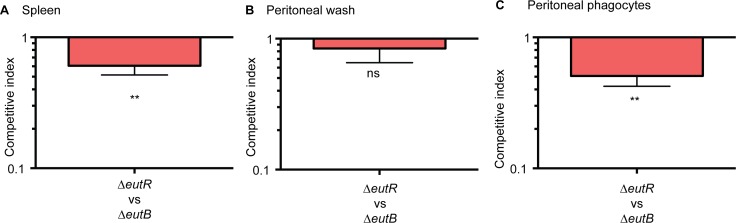
EutR promotes recognition and adaptation to the intracellular environment. (**A-C**) Competition assays between Δ*eutR*::Cm^R^ (CJA007) and Δ*eutB* (CJA020) strains collected from (**A**) harvested spleens, (**B**) the peritoneal cavity, or (**C**) phagocytized cells at 6 h pi. Mice were intraperitoneally infected with 1:1 mixtures of the Δ*eutR* and Δ*eutB* strains. Each column represents a CI. Each column shows the geometric mean value ± SE for each group (n = 2 litters (6–8 animals)). *, *P* ≤ 0.05; **, *P* ≤ 0.005; ***, *P* ≤ 0.0005; *P* > 0.05 = ns.

Next, we confirmed that EutR mediates dissemination specifically through intracellular survival *in vivo*. To further discriminate between ethanolamine-associated signaling and ethanolamine metabolism, we infected mice with equal numbers of the Δ*eutR*::Cm^R^ and Δ*eutB* strains by intraperitoneal injection. At 6 h pi, the Δ*eutR* strain was recovered at significantly lower numbers compared to the Δ*eutB* strain from the spleen (Fig 6A). Furthermore, we assessed bacterial burden in the peritoneal cavity. At this site, there were no significant differences between the Δ*eutR* and Δ*eutB* strains in the total bacteria recovered (Fig 6B), the majority of which were extracellular (S10 Fig). However, the Δ*eutR* strain was recovered at significantly lower numbers compared to the Δ*eutB* strain in the phagocytized population of *S*. Typhimurium within the peritoneal cavity (Fig 6C). These findings reveal that EutR augments S. Typhimurium fitness during systemic infection. Our findings differ from a previous study that used a genetic screen to identify genes important for systemic virulence [43]. Discrepancies may reflect differences in study design such as the age and genetic background of mice, route of infection, and/or duration of infection. Importantly, using *in vitro* and *in vivo* approaches, our data establish a genetic and functional role for EutR in *S*. Typhimurium systemic disease, and altogether, these results indicate that EutR contributes to the ability of *S*. Typhimurium to gauge and adapt to the intracellular environment *in vivo*.

### EutR signaling during systemic infection

**Fig 7:**
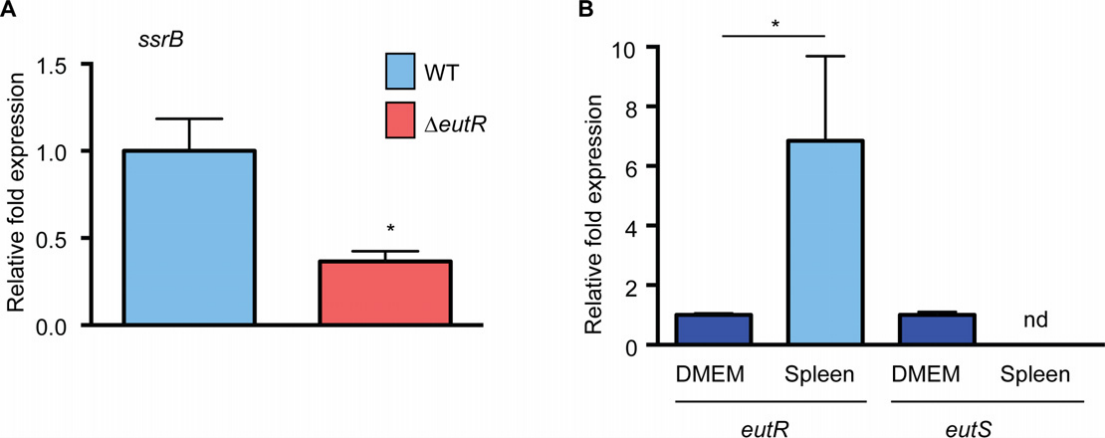
EutR-associated signaling *in vivo*. (**A**) qRT-PCR analysis of *ssrB* expression in WT *S*. Typhimurium (SL1344) or the Δ*eutR* strain (CJA009) harvested from infected spleens. (**B**) qRT-PCR analysis of *eutR* or *eutS* expression in WT *S*. Typhimurium (SL1344) harvested from infected spleens compared to *S*. Typhimurium (SL1344) grown in tissue culture medium (DMEM). For (**A**) and (**B**), n = 2–3; error bars represent the geometric mean ± SD; *strB* was used as the endogenous control. *, *P* ≤ 0.05. nd = not detected.

The *in vitro* studies identified targets of EutR-dependent gene regulation. To test our findings within the complexities of the *in vivo* environment, we assessed EutR-dependent regulation of *ssrB* using single strain infections and purified *S*. Typhimurium RNA from harvested spleens. Expression of *ssrB* was significantly decreased in the Δ*eutR* strain compared to WT (Fig 7A and S11 Fig), which is consistent with the data presented in Fig 4B. Additionally, we measured expression of *eutR* and *eutS* in WT *S*. Typhimurium recovered from the spleen relative to *S*. Typhimurium grown *in vitro*. Notably, *eutR* expression was significantly increased in the spleen, whereas expression of *eutS* was not detectable (Fig 7B). These data further highlight the dynamic role of EutR in S. Typhimurium pathogenesis from driving ethanolamine metabolism in the intestine to promoting virulence gene expression in later stages of disease.

### Conclusions

**Fig 8:**
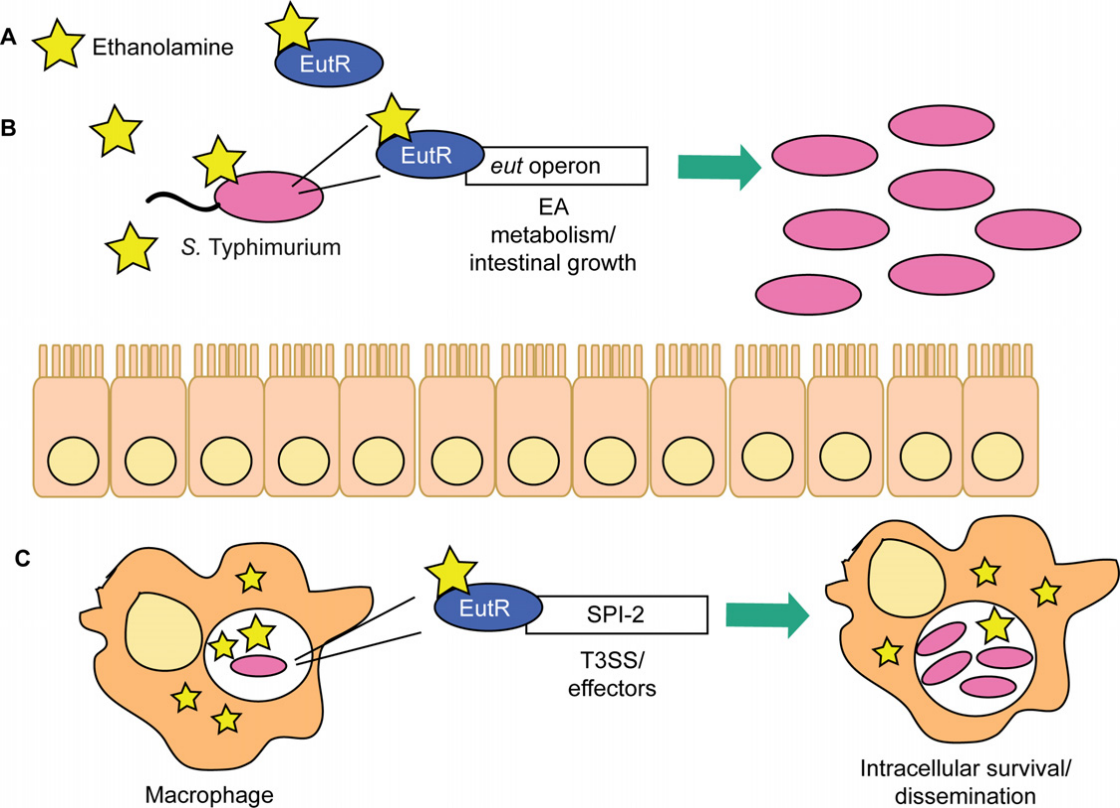
EutR in *S*. Typhimurium niche adaptation. (**A**) EutR senses ethanolamine to activate transcription. (**B**) In the intestine, EutR promotes expression of the *eut* operon that encodes ethanolamine metabolism, thereby enhancing *S*. Typhimurium growth. (**C**) EutR expression in macrophages activates expression of genes in SPI-2, which are required for intramacrophage survival and dissemination.

These findings reveal a novel signaling pathway critical for *S*. Typhimurium to enhance disease progression during infection. We propose a model in which *S*. Typhimurium relies on ethanolamine signaling through EutR to gauge distinct environments in the host and then modulate expression of genes encoding metabolism and virulence (Fig 8). The resident microbiota do not readily metabolize ethanolamine [33]. Thus, to establish infection, *S*. Typhimurium sidesteps nutritional competition by respiring ethanolamine in conjunction with tetrathionate, an electron acceptor generated specifically during intestinal inflammation [17,44]. Fermentation of ethanolamine provides very little growth [17]; hence, outside of the intestine, and in the absence of bacterial competition, *S*. Typhimurium preferentially utilizes alternative nutrients [23]. This enables EutR to direct expression of traits necessary for dissemination and systemic infection. Additional experiments are necessary to determine what factors influence the transition from driving metabolism to influencing virulence in the intestine.

Genes encoding ethanolamine utilization are widespread in pathogenic bacteria as well as in members of the resident microbiota [45], and the extracellular pathogen EHEC responds to ethanolamine to regulate virulence gene expression [22]. Therefore, ethanolamine signaling may be a conserved strategy used by diverse pathogens to coordinate metabolism and virulence in response to distinct host environments. Our findings highlight a sophisticated mechanism in which *S*. Typhimurium exploits an abundant and essential molecule within the host to gain specific information about the localized environment and modulate gene expression to overcome bacterial and host resistance mechanisms.

## Materials and Methods

### Strains and plasmids

All strains and plasmids used in this study are listed in S1 Table. Luria-Bertani (LB), Dulbecco’s Modified Eagle Medium (DMEM) (Invitrogen), or minimal medium (described below) were used as indicated. Ethanolamine (Sigma) and/or vitamin B_12_ (Sigma) were supplemented to the media as indicated in the main text. Unless indicated otherwise, 150 nM vitamin B_12_ was added whenever ethanolamine was added to the growth medium. Antibiotics were used in the following concentrations: ampicillin (100μg/ml), streptomycin (100μg/ml), chloramphenicol (20μg/ml), and kanamycin (50μg/ml). Recombinant DNA and molecular biology techniques were performed as described previously [46].

### Construction of isogenic mutants

*S*. Typhimurium SL1344 [47] and its derivatives were used in all experiments. The *invG* mutant (strain AJK61) was a gift from James Casanova and was constructed as previously described [48]. Nonpolar *eutR* and *eutB* deletion strains were generated in WT and Δ*invG* backgrounds using λ-red mutagenesis [49]. Briefly, PCR products (obtained with primers listed in S2 Table) were amplified from plasmid pKD3 or pKD4 with flanking regions matching *eutR* or *eutB* and then transformed into *S*. Typhimurium expressing the Red genes from plasmid pKD46. The resistance cassette was resolved with flippase from temperature-sensitive plasmid pCP20, which was then cured through growth at 42°C. Unresolved strains were used in the murine competition assays as indicated in the text and figure legends. All deletions were confirmed by sequencing. The *eutR* mutant was complemented with pCJA002. Plasmid pCJA002 was constructed by amplifying *S*. Typhimurium genomic DNA using primers specific to the *eutR* gene, including 206 nucleotides upstream of the ATG start site (listed in S2 Table). Amplified DNA was digested with HindIII and BamHI and inserted into pGEN-MCS [50] (Addgene MTA). As controls, WT and the Δ*eutR* strains were transformed with empty pGEN-MCS vectors for use in the complementation experiments. The EutR::Flag strain was generated as described for the deletion strains, using pSUB11 as described [51].

### Culture conditions for growth curves and gene expression analyses

All cultures were grown overnight in LB and then diluted 1:100 in the indicated medium and grown at 37°C. For RNA expression studies, cultures were grown until mid-logarithmic phase (OD_600_ = 0.45–0.55). Cultures grown in DMEM were incubated statically under a 5% CO_2_ atmosphere (to mimic tissue culture conditions). SPI-2 inducing medium was prepared as previously described (100mM Bis/Tris-HCl pH 7.0, 5mM KCl, 7.5mM (NH_4_)_2_SO_4_, 0.5mM K_2_SO_4_, 1mM KH_2_PO_4_, 38mM glycerol, 0.1% casamino acids, and 8μM MgCl_2_) [32], and cultures were grown aerobically with agitation [32].

### RNA extraction and quantitative reverse transcription polymerase chain reaction (qRT-PCR)

For the *in vitro* studies, RNA was extracted from *S*. Typhimurium cells grown in culture medium as described or from phagocytized *S*. Typhimurium. Cells were resuspended in Trizol (Life Technologies) and RNA was purified using the RiboPure kit (Ambion). For the *in vivo* studies, spleens were harvested at 2 dpi and homogenized in 1 mL Trizol per 100 mg tissue [52]. RNA was isolated using standard molecular biological procedures. Primer validation and qRT-PCR was performed as described previously [53] using primers listed in S2 Table. Briefly, RNA was extracted from three biological replicates, and qRT-PCR was performed in a one-step reaction using an ABI 7500 sequence detection system (Applied Biosystems). Data were collected using the ABI software Detection 1.2 software (Applied Biosystems). All data were normalized to the endogenous control *strB* (main text) or to 16S rRNA (RNA was diluted 1:1000) as previously performed [54,55]. Controls were used as indicated in figure legends and analyzed using the comparative critical threshold (C_*T*_) method. The Student’s unpaired *t* test was used to determine statistical significance.

### Tissue culture

RAW, J774, and HeLa cells were routinely cultured in DMEM supplemented with 10% FBS and 1x penicillin-streptomycin–glutamine; peritoneal exudate macrophages (PEMs) were cultured in RPMI 1640 supplemented with 10% FBS, 20% L-929 conditioned medium, and 1x penicillin-streptomycin–glutamine. PEMs were isolated as described [56]. Antibiotics were omitted during bacterial infections.

For epithelial cell infection bacterial cultures were grown under invasion-inducing conditions [27]. Briefly, overnight cultures were diluted back 1:100 and grown without agitation in LB until late logarithmic phase (OD_600_ of approximately 1.0) at 37°C. Bacterial cells were washed and resuspended in 1x phosphate buffered saline before infection. HeLa cells were placed in DMEM or DMEM supplemented with 5 mM ethanolamine and 150 nM vitamin B_12_. HeLa cells were infected at a multiplicity of infection (MOI) of 100 for 1 h and either lysed directly or treated with 100 μg/ml gentamicin for 30 min to kill any extracellular bacteria. Percent invasion was calculated as the number of intracellular bacteria as a percent of the directly lysed sample and normalized such that wild type was equal to 100%.

For macrophage assays, we used an *invG* mutant (deficient in cell invasion) as the WT strain, and we generated corresponding Δ*eutRΔinvG* and Δ*eutBΔinvG* strains (described above). These strains were used because invasive *S*. Typhimurium rapidly kills macrophages [57]. Additionally, expression of invasion-associated genes are down-regulated after entry into host cells; therefore, this strain more closely mimics *S*. Typhimurium as it is encountered by professional phagocytes after penetration of the epithelial barrier [58].

Gentamicin protection assays were performed according to published methods [14,57,59,60]. *S*. Typhimurium was grown overnight in LB, washed and re-suspended in PBS before incubation with macrophages (without the addition of ethanolamine or vitamin B_12_) at an MOI of 50. After 30 min of incubation, extracellular bacteria were killed with 100 μg/ml gentamicin treatment for 30 min, before replacement with media containing 10 μg/ml gentamicin for the remainder of the assay. Cells were lysed at indicated time points in 1% Triton-X and colony forming units (cfu) determined by serial dilutions and plating onto LB agar. After internalization, cells were treated with gentamicin and lysed to enumerate viable intracellular bacteria at time 0 h. Survival was calculated as previously described [14]. Briefly, the viable cfu at the indicated time points were determined as the percentage of this intracellular time 0 h population and normalized such that wild type was equal to 100%. For all assays, the Student *t* test was used to determine statistical significance.

### Mouse studies

All experiments were approved by the Institutional Animal Care and Use Committee at the University of Virginia School of Medicine. For the colitis infections, female C57BL/6 (10–12 week old) mice were given a single dose of 20 mg streptomycin 24 h prior to infection [61]. Mice were infected with an equal mixture of 5 x 10^8^ cfu of the indicated strains. Fresh fecal pellets were collected daily, and mice were euthanized at 4 dpi to assess bacterial burden in the colon and spleen. Tissue samples were weighed, homogenized in 1 ml PBS, and bacterial numbers were quantified by plating serial dilutions of homogenates on MacConkey agar supplemented with streptomycin or with chloramphenicol. The competitive index was calculated as the ratio of Δ*eutR* to wild type (WT) or Δ*eutB* strains or the ratio of the Δ*eutB* to WT recovered normalized to the ratio in the inoculum. Statistical significance was determined by one-sample *t* test with an expected value of 1. Comparisons between competitive indexes were performed using the Mann-Whitney *U* test.

For the systemic competition experiments, mice were infected intraperitoneally (i.p.) with 1x10^5^ cfu of the Δ*eutR* and Δ*eutB* strains. Spleens were harvested at 6 h and bacterial burden was assessed as described above. Bacterial burden in the peritoneal cavity was assessed as described [62]. Briefly, following euthanasia, 5mL of PBS was injected into the peritoneal cavity, aspirated, and immediately placed on ice. Samples were split into two aliquots, one receiving 100 μg/ml gentamicin treatment. After 30 minutes on ice, samples were washed, lysed and plated as described above. For RNA analyses, mice were infected by i.p. with 1x10^4^ cfu of WT or the Δ*eutR* strain, and spleens were harvested at 2 dpi.

### Electrophoretic mobility shift assays (EMSAs)

Plasmid pDC24 or the empty vector pMAL-c5X was used for the EMSA assays. This plasmid was constructed by amplifying the *eutR* gene from the *S*. Typhimurium strain SL1344 with indicated primers (S2 Table). The resulting PCR product was cloned into the Nco1/Sbf1 cloning site of vector pMAL-c5X. EutR was purified under native conditions as described [19]. Briefly, the MBP-tagged EutR protein was purified by growing the *E*. *coli* strain NEBexpress cells (NEB) containing pDC24 at 37°C in LB with glucose (0.2% final concentration) and ampicillin (100 μg/ml) to an OD_600_ of 0.5, at which point IPTG was added to a final concentration of 0.3 mM and allowed to induce overnight at 18°C. Cells were harvested by centrifugation at 4000 x *g* for 20 min and then resuspended in 25 mL column buffer (20 mM Tris-HCl; 200 mM NaCl; 1 mM EDTA) and lysed by homogenization. The lysed cells were centrifuged, and the lysate was loaded onto a gravity column (Qiagen) with amylose resin. The column was washed with column buffer and then eluted with column buffer containing 10 mM maltose. Fractions containing purified proteins were confirmed by SDS-PAGE and Western analysis, and the protein concentration was determined using a NanoDrop Spectrophotometer. PCR-amplified DNA probes (listed in text and described in S2 Table) were generated as previously described [19,63]. DNA probes were end-labeled with [γ-32^P^]-ATP (Perkin-Elmer) using T4 polynucleotide kinase (NEB) following standard procedures [64]. End-labeled fragments were run on a 6% polyacrylamide gel, excised, and purified using the Qiagen PCR purification kit.

EMSAs were performed by adding purified EutR-MBP or MBP to labeled DNA in binding buffer (500 μg ml^-1^ BSA (NEB), 50 ng poly-dIdC, 60 mM HEPES pH 7.5, 5 mM EDTA, 3 mM dithiothreitol (DTT), 300 mM KCl, and 25 mM MgCl_2_). Ethanolamine (1 mM) and vitamin nM B_12_ (150 nM) were added to the reactions. Reactions were incubated for 25 minutes at 25°C. Then, a 1% Ficoll solution was added to the reactions immediately before loading the samples on the gel. The reactions were electrophoresed for approximately 6 h at 150 V on a 6% polyacrylamide gel, dried, and imaged with a phosphorimager (Molecular Dynamics).

### Chromatin immunoprecipitation (ChIP) and ChIP qPCR

ChIP was performed using an WT *S*. Typhimurium (untagged EutR) or with the *S*. Typhimurium *eutR* mutant transformed with the EutR::MBP plasmid. Strains were grown in DMEM supplemented with 5 mM EA, 150 nM B_12_, and 0.5 μM IPTG until cells reached an OD_600_ of approximately 0.8. Cross-linking and ChIP were performed based on established methods [65]. Formaldehyde was added (1% final concentration) for cross-linking, and cells were incubated at room temperature for 20 min. Reactions were quenched with 0.5 M glycine, then samples were pelleted, resuspended in TBS, and washed. Cells were lysed with 2 mg/ml lysozyme and incubated at 37°C for 30 min. Subsequently, samples were placed on ice and sonicated. Insoluble cell debris was removed by centrifugation, and supernatants were saved. Immunoprecipitation was carried out by incubating samples with amylose beads (NEB) in buffer for 2 h at 4°C with gentle mixing. Beads were pelleted and washed. Then the samples were incubated for 10 min at 65°C in elution buffer with occasional gentle mixing. Samples were centrifuged and supernatants were collected. To reverse the cross-link, samples were boiled for 10 min and DNA was purified using the Qiagen PCR purification kit. For ChIP-quantitative PCR (qPCR) experiments, untreated chromatin was de-cross-linked by boiling for 10 min and purified, for use as the “input” control. Primers amplifying the *strB* gene were used as the negative control. The fold enrichment of each promoter represents the value of the immunoprecipitated DNA divided by the input unprecipitated DNA [66,67]. These values were normalized to the values obtained for each promoter precipitated using untagged EutR in order to account for non-specific enrichment.

### Accession numbers for genes/proteins mentioned in text

*eutR*, NP_461389; *eutS*, NP_461405; *eutB*, NP_461393; *sipC*, NP_461805; *ssrA*, NP_460357; *ssrB*, NP_460356; *ssaB*, NP_460358; *sseA*, NP_460362; *ssaG*, NP_460371; *sifA*, NP_460194.

## Supporting Information

S1 Fig qRT-PCR of *sipC* from WT *S*. Typhimurium (SL1344) grown in LB or LB supplemented with 5 mM ethanolamine (EA). n = 3; error bars represent the geometric mean ± SD.16S rRNA was used as the endogenous control. **, *P* ≤ 0.005.
(TIF)

S2 Fig qRT-PCR analysis of *ssrB* expression in WT *S*. Typhimurium (SL1344) grown in DMEM or SPI-2 inducing medium. n = 3; error bars represent the geometric mean ± SD; *strB* was used as the endogenous control. ***, *P* ≤ 0.0005.
(TIF)

S3 Fig Expression of chromosomal EutR::Flag in response to varying ethanolamine concentrations. RpoA is shown as a loading control.
(TIF)

S4 Fig qRT-PCR analysis of *ssrB* expression in WT *S*. Typhimurium (SL1344) grown in LB or LB supplemented with 5 mM ethanolamine (EA). n = 3; error bars represent the geometric mean ± SD; *strB* was used as the endogenous control. *P* > 0.05 = ns.
(TIF)

S5 Fig qRT-PCR of *eutR* from RNA isolated from *S*. Typhimurium (AJK61) grown in DMEM or after 5 h phagocytosis in J774 macrophages. Statistical significance relative to cells grown in DMEM is indicated. n = 3; error bars represent the geometric mean ± SD; *strB* was used as the endogenous control.*, *P* ≤ 0.05.
(TIF)

S6 Fig (A) qRT-PCR of *eutR* and *eutS* from RNA isolated from the *S*. Typhimurium (SL1344) grown in SPI-2 inducing medium with ethanolamine (EA) supplementation as indicated. (B) qRT-PCR of *eutR* and *eutS* from RNA isolated from the *S*. Typhimurium (SL1344) grown in DMEM with EA supplementation as indicated. Statistical significance is shown relative to cells grown without EA supplementation. n = 3; error bars represent the geometric mean ± SD; *strB* was used as the endogenous control.***, *P* ≤ 0.0005.
(TIF)

S7 Fig qRT-PCR analysis of *ssrB* from RNA isolated from WT (AJK61) or the Δ*eutR* (CJA023) *S*. Typhimurium strains after 5 h phagocytosis in J774 macrophages. n = 3; error bars represent the geometric mean ± SD; *strB* was used as the endogenous control. **, *P* ≤ 0.005.
(TIF)

S8 Fig qRT-PCR analysis of SPI-2-encoded and associated (*sifA*) genes from RNA isolated from *S*. Typhimurium (AJK61) or the Δ*eutR* (CJA023) strains after 5 h phagocytosis in RAW macrophages. n = 3; error bars represent the geometric mean ± SD; 16S rRNA was used as the endogenous control. *, *P* ≤ 0.05; *P* > 0.05 = ns.
(TIF)

S9 Fig Intramacrophage replication of *S*. Typhimurium WT (AJK61) and Δ*eutR* (CJA023) after 7 h phagocytosis in J774 macrophages. Error bars represent the geometric mean ± SE of six independent experiments; **, *P* ≤ 0.005.
(TIF)

S10 Fig Total bacteria recovered from the peritoneal cavity. Mice were i.p. infected with equal numbers of Δ*eutR* (CJA007) and Δ*eutB* (CJA020) *S*. Typhimurium strains. After 6 h, peritoneal fluid was harvested and plated to determine total bacterial burden or treated with gentamicin and then plated to determine phagocytized bacterial burden (n = 2 litters (6–8 animals)).
(TIF)

S11 Fig qRT-PCR analysis of *ssrB* expression in WT *S*. Typhimurium (SL1344) or the Δ*eutR* strain (CJA009) harvested from infected spleens. n = 3; error bars represent the geometric mean ± SD. 16S rRNA was used as the endogenous control. *, *P* ≤ 0.05.
(TIF)

S1 Table Bacterial strains and plasmids. (PDF)

S2 Table Oligonucleotide primers. (PDF)
