# Correction: Ethanolamine Signaling Promotes *Salmonella* Niche Recognition and Adaptation during Infection

**DOI:** 10.1371/journal.ppat.1005365

**Published:** 2015-12-18

**Authors:** Christopher J. Anderson, David E. Clark, Mazhar Adli, Melissa M. Kendall

There is a grant number missing from the Funding Statement. Please see the corrected Funding Statement here.

This work is funded by the National Institutes of Health, National Institute of Allergy and Infectious Diseases (AI118732) to MMK and the training grant (5T32AI007046) to CJA. The funders had no role in study design, data collection and analysis, decision to publish, or preparation of the manuscript.

## References

[1] Anderson CJ, Clark DE, Adli M, Kendall MM (2015) Ethanolamine Signaling Promotes *Salmonella* Niche Recognition and Adaptation during Infection. PLoS Pathog 11(11): e1005278 doi:10.1371/journal.ppat.1005278 26565973 10.1371/journal.ppat.1005278 PMC4643982

