# Supplementary figures and images for: Ethanolamine Signaling Promotes Salmonella Niche Recognition and Adaptation during Infection
Source: PLoS Pathog. 2015 Nov 13;11(11):e1005278. doi: 10.1371/journal.ppat.1005278 (PMC4643982; doi:10.1371/journal.ppat.1005278)

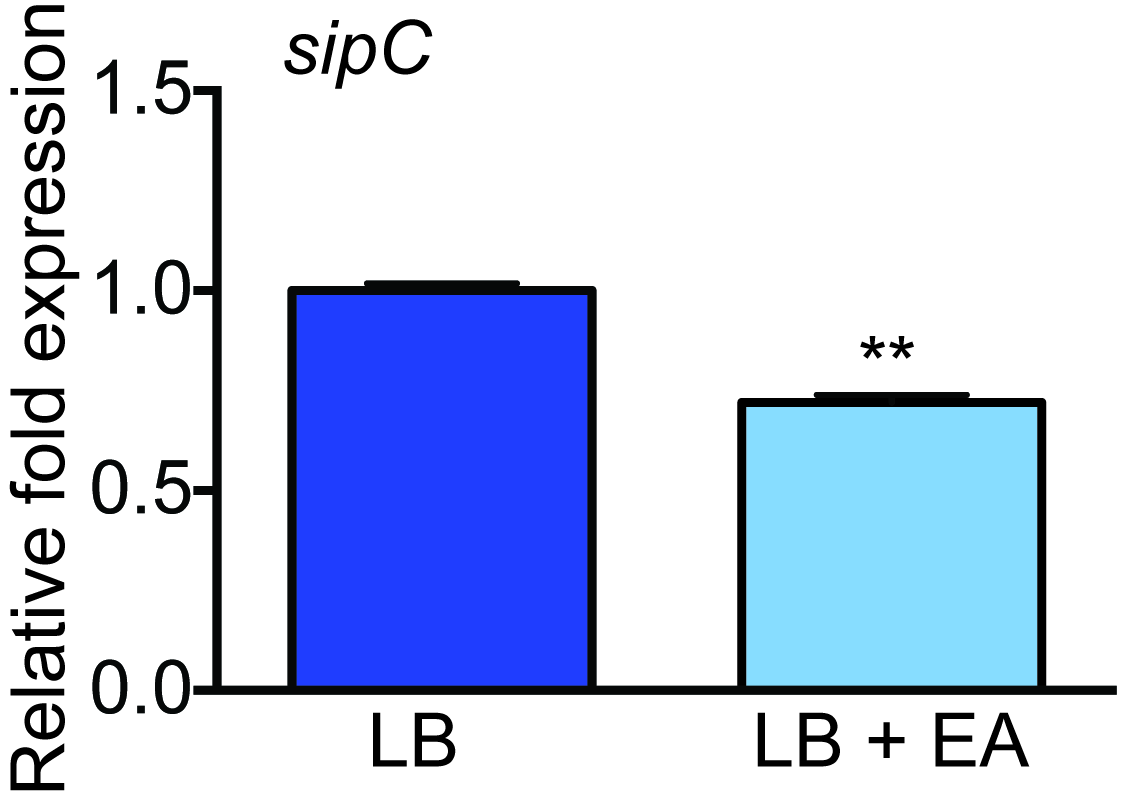

Supplement: S1 Fig — n = 3; error bars represent the geometric mean ± SD.16S rRNA was used as the endogenous control. **, P ≤ 0.005. (TIF) [file ppat.1005278.s001.tif]

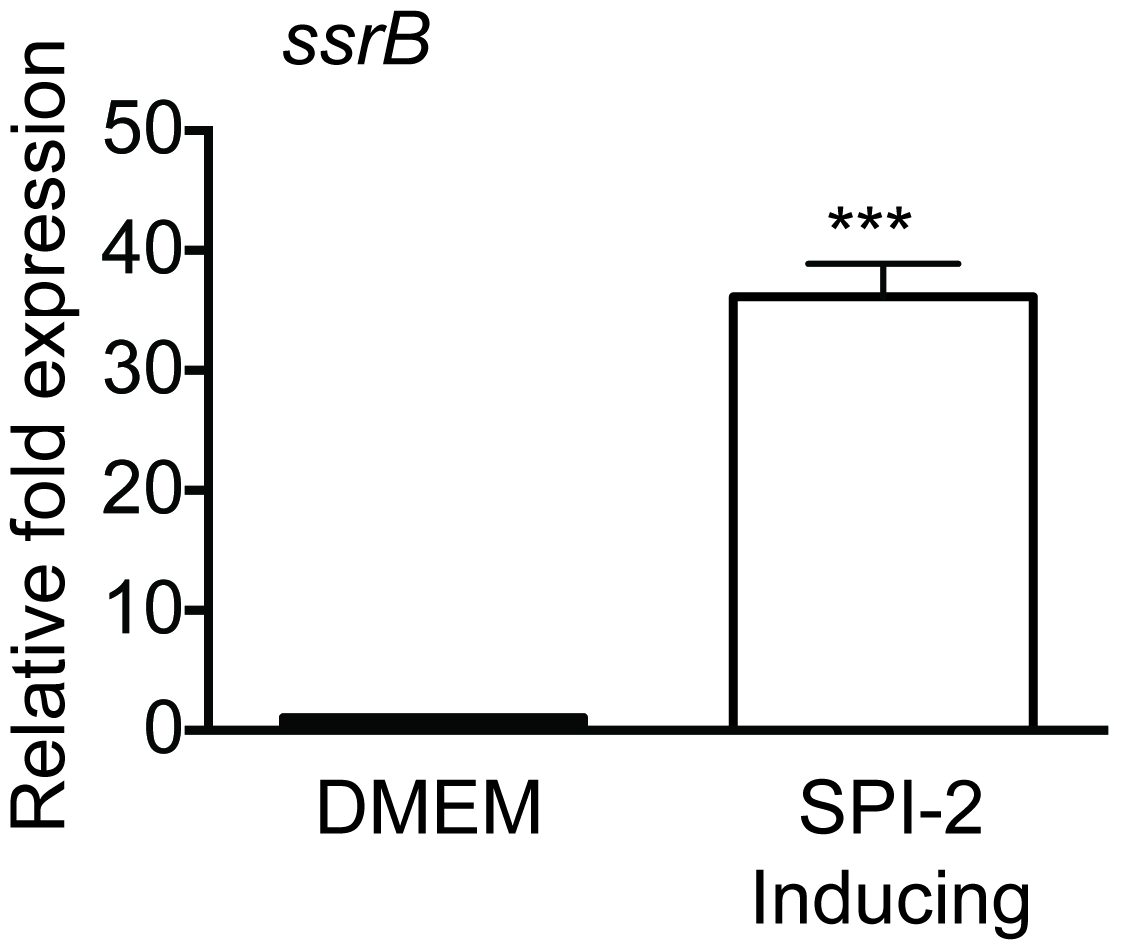

Supplement: S2 Fig — n = 3; error bars represent the geometric mean ± SD; strB was used as the endogenous control. ***, P ≤ 0.0005. (TIF) [file ppat.1005278.s002.tif]

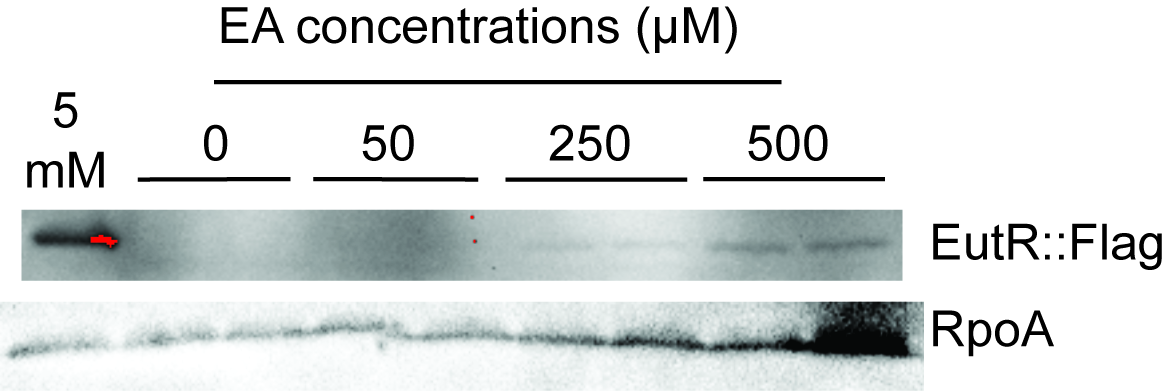

Supplement: S3 Fig — RpoA is shown as a loading control. (TIF) [file ppat.1005278.s003.tif]

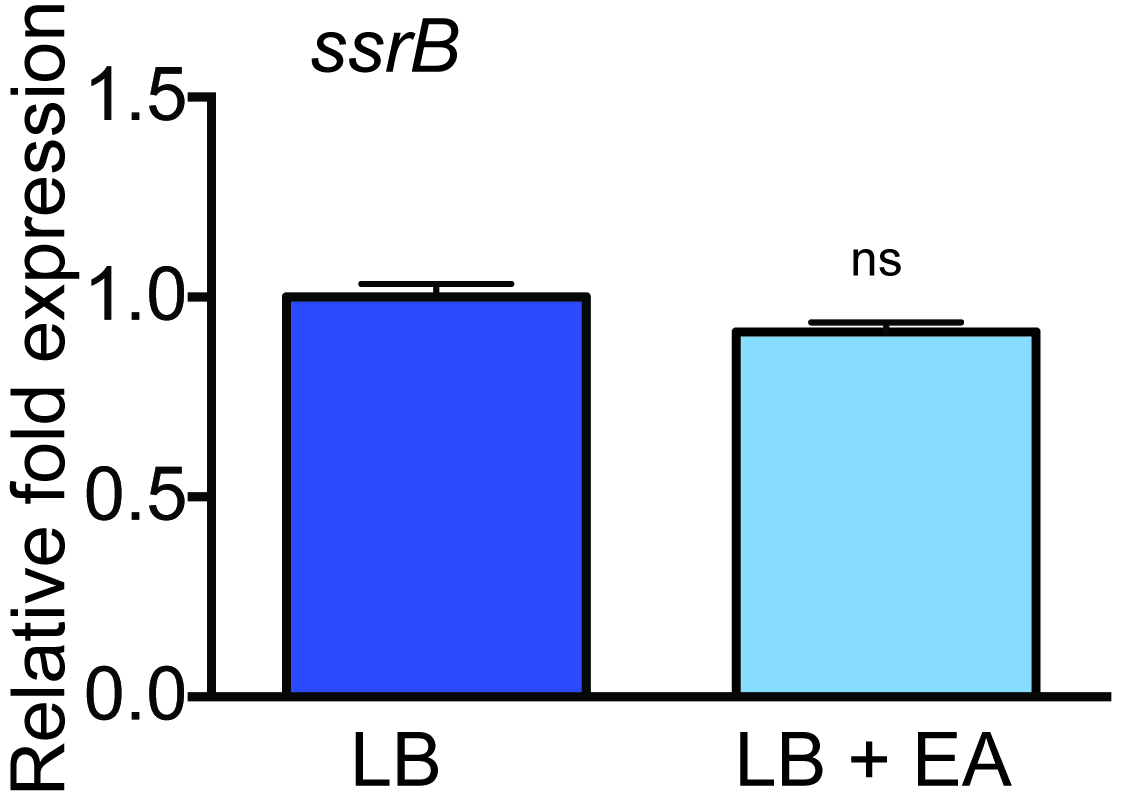

Supplement: S4 Fig — n = 3; error bars represent the geometric mean ± SD; strB was used as the endogenous control. P > 0.05 = ns. (TIF) [file ppat.1005278.s004.tif]

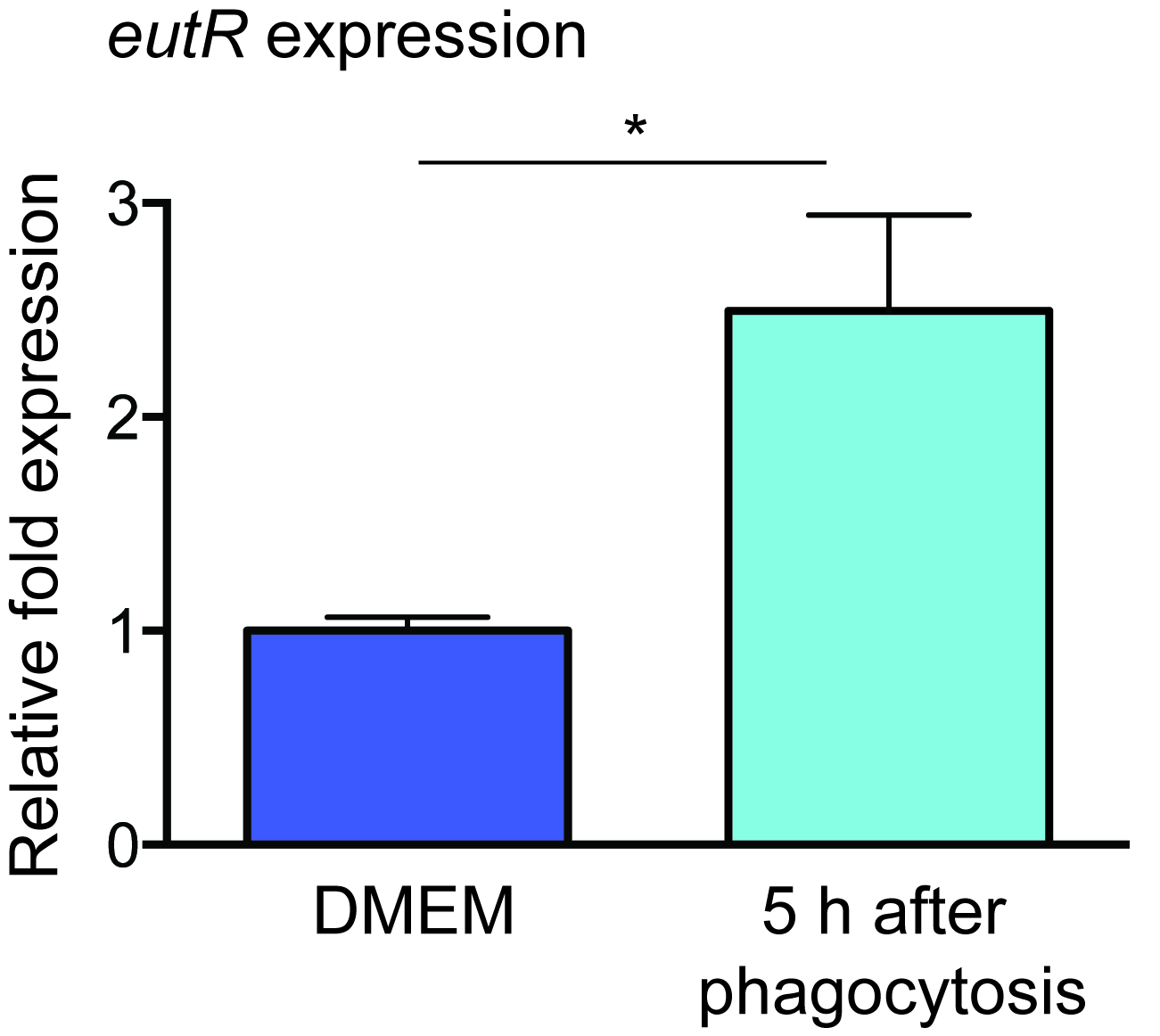

Supplement: S5 Fig — Statistical significance relative to cells grown in DMEM is indicated. n = 3; error bars represent the geometric mean ± SD; strB was used as the endogenous control.*, P ≤ 0.05. (TIF) [file ppat.1005278.s005.tif]

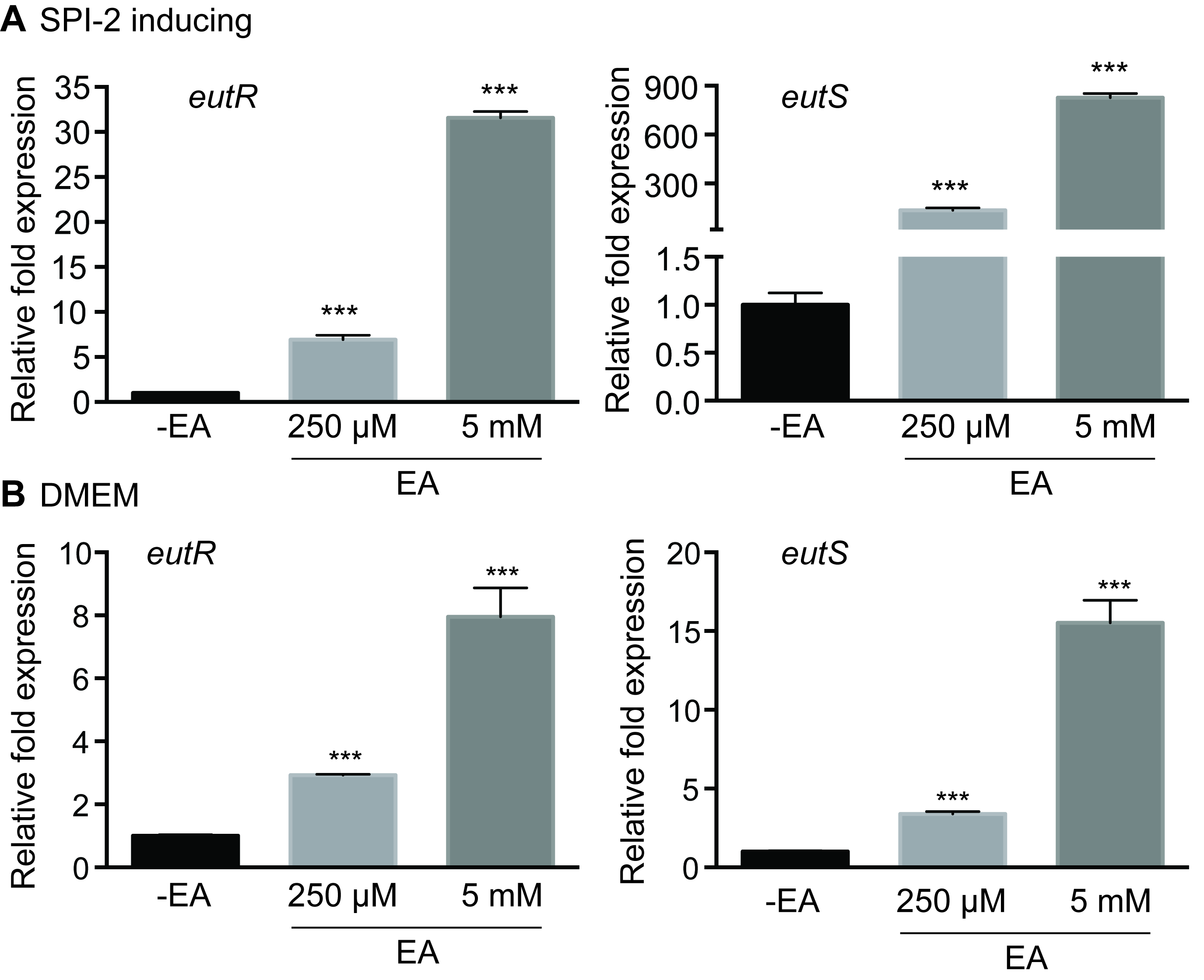

Supplement: S6 Fig — Statistical significance is shown relative to cells grown without EA supplementation. n = 3; error bars represent the geometric mean ± SD; strB was used as the endogenous control.***, P ≤ 0.0005. (TIF) [file ppat.1005278.s006.tif]

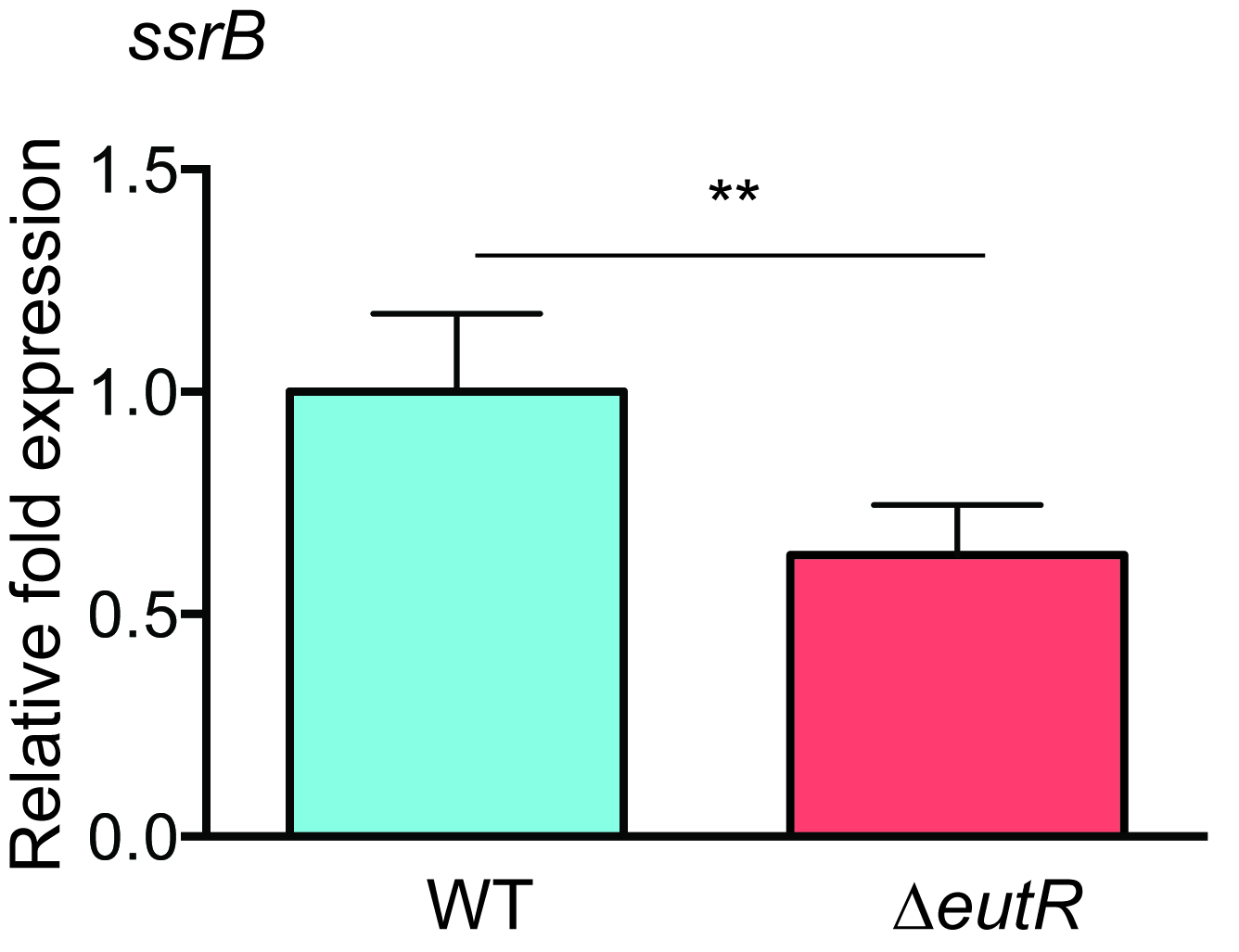

Supplement: S7 Fig — n = 3; error bars represent the geometric mean ± SD; strB was used as the endogenous control. **, P ≤ 0.005. (TIF) [file ppat.1005278.s007.tif]

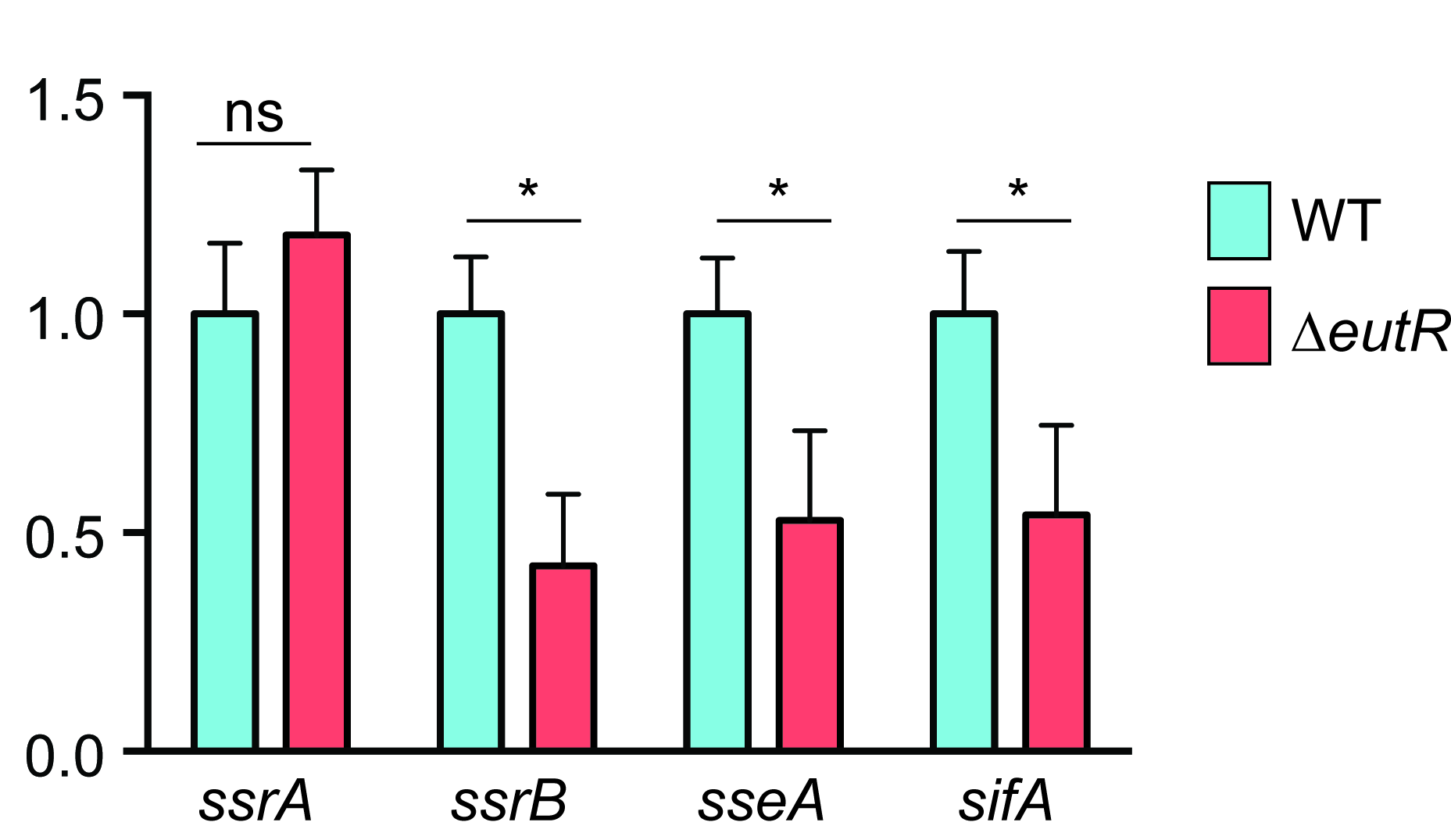

Supplement: S8 Fig — n = 3; error bars represent the geometric mean ± SD; 16S rRNA was used as the endogenous control. *, P ≤ 0.05; P > 0.05 = ns. (TIF) [file ppat.1005278.s008.tif]

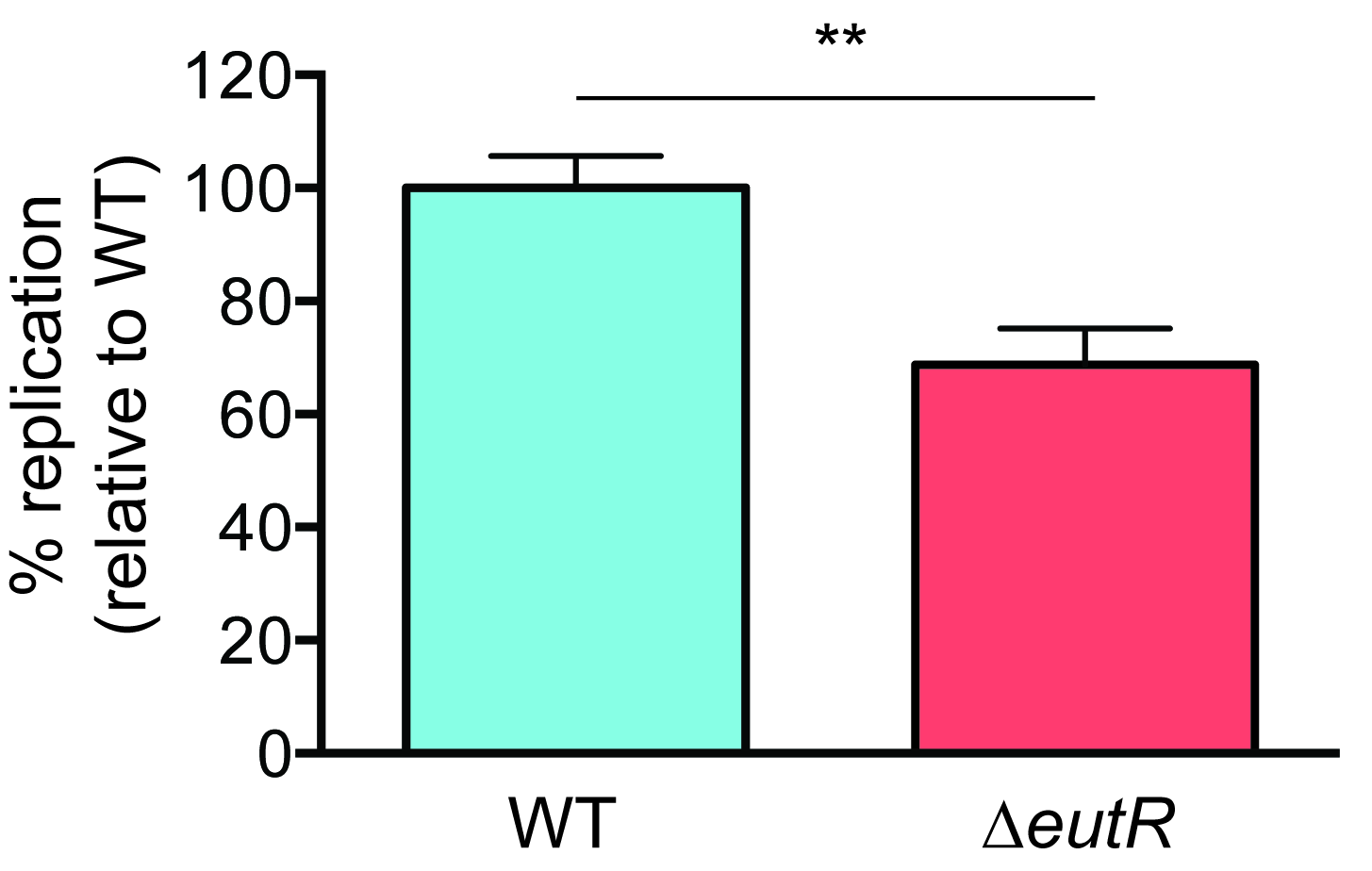

Supplement: S9 Fig — Error bars represent the geometric mean ± SE of six independent experiments; **, P ≤ 0.005. (TIF) [file ppat.1005278.s009.tif]

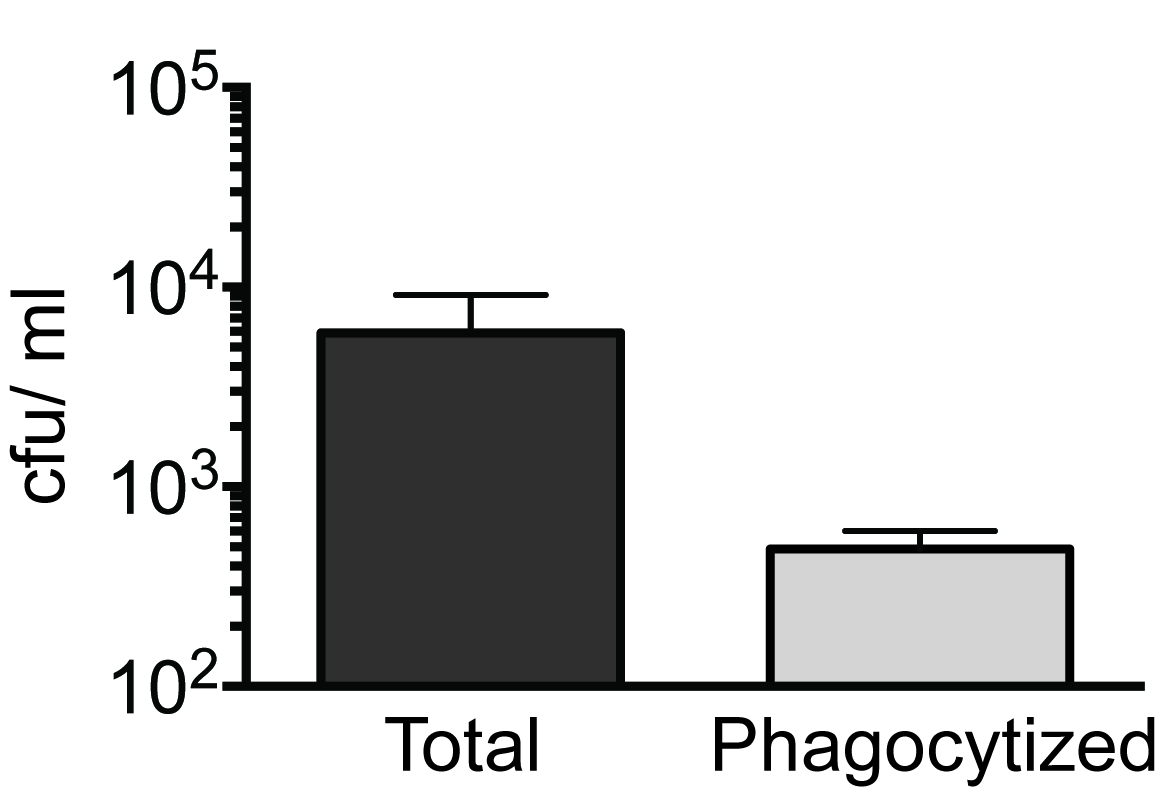

Supplement: S10 Fig — Mice were i.p. infected with equal numbers of ΔeutR (CJA007) and ΔeutB (CJA020) S. Typhimurium strains. After 6 h, peritoneal fluid was harvested and plated to determine total bacterial burden or treated with gentamicin and then plated to determine phagocytized bacterial burden (n = 2 litters (6–8 animals)). (TIF) [file ppat.1005278.s010.tif]

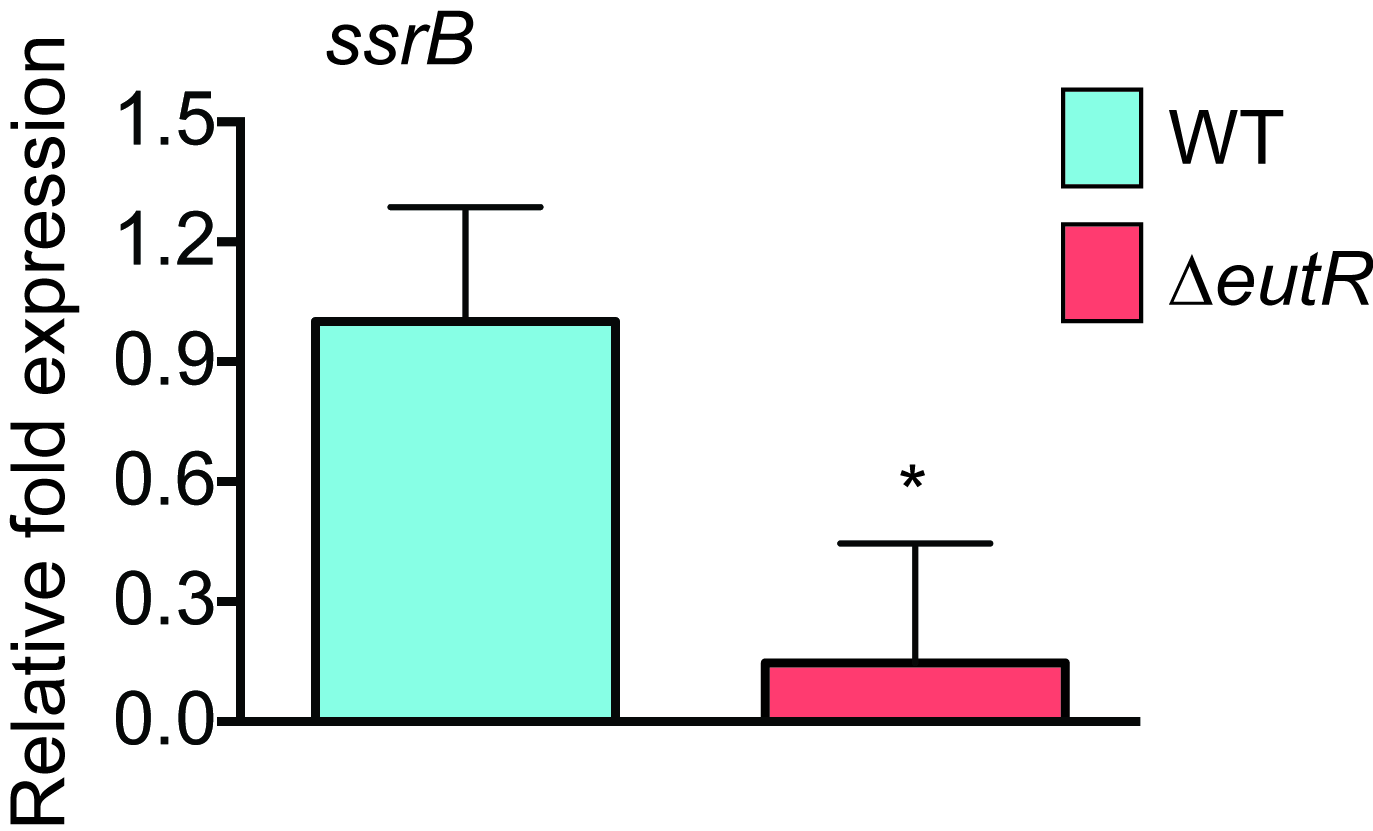

Supplement: S11 Fig — n = 3; error bars represent the geometric mean ± SD. 16S rRNA was used as the endogenous control. *, P ≤ 0.05. (TIF) [file ppat.1005278.s011.tif]
