# Supplementary material for: Ethanolamine Signaling Promotes Salmonella Niche Recognition and Adaptation during Infection
Source: PLoS Pathog. 2015 Nov 13;11(11):e1005278. doi: 10.1371/journal.ppat.1005278 (PMC4643982; doi:10.1371/journal.ppat.1005278)
Supplement: S1 Table — (PDF) [file ppat.1005278.s012.pdf]

**Table S1. Bacterial strains and plasmids**			
**Designation**	**Genotype and relevant characteristics**	**Antibiotic Resistance**	**Reference**
SL1344	Wild type	Streptomycin	[1]
CJA007	SL1344 Δ*eutR*:Chl (chloramphenicol resistance cassette replaces the *eutR* gene)	Streptomycin Chloramphenicol	Present study
CJA009	SL1344 Δ*eutR*	Streptomycin	Present study
CJA018	SL1344 Δ*eutB*:Chl (chloramphenicol resistance cassette replaces the *eutB* gene)	Streptomycin Chloramphenicol	
CJA020	SL1344 Δ*eutB*	Streptomycin	Present study
AJK61	SL1344 Δ*invG*	Streptomycin	[2]
CJA023	SL1344 Δ*invG*Δ*eutR*	Streptomycin	Present study
CJA028	SL1344 Δ*invG*Δ*eutB*	Streptomycin	Present study
CJA032	SL1344 Δ*invG*Δ*eutR* + pGEN	Streptomycin Ampicillin	Present study
CJA033	SL1344 Δ*invG*Δ*eutR* + pCJA002	Streptomycin Ampicillin	Present study
CJA034	SL1344 Δ*invG* + pGEN	Streptomycin Ampicillin	Present study
CJA037	SL1344 Δ*invG* EutR::FLAG	Streptomycin	Present study
DC26	SL1344 Δ*eutR* + pDC24	Streptomycin Ampicillin	Present study
**Plasmids**			
pGEN	Cloning vector	Ampicillin	[3]
pMAL-c5X	Cloning vector	Ampicillin	New England Biolabs
pCJA002	pGEN + *eutR*	Ampicillin	Present study
pDC24	SL1344 *eutR* in pMAL-c5X	Ampicillin	Present study
pKD46		Ampicillin	[4]
pKD3		Chloramphenicol	[4]
pKD4		Kanamycin	[4]
pCP20		Ampicillin Chloramphenicol	[4]
pSUB11		Ampicillin	[5]

1. Hoiseth SK, Stocker BA (1982) Aromatic-dependent *Salmonella typhimurium* are non-virulenct and effective as live vaccines. Nature 291: 238-239.
2. Criss AK, Ahlgren DM, Jou TS, McCormick BA, Casanova JE (2001) The GTPase Rac1 selectively regulates *Salmonella* invasion at the apical plasma membrane of polarized epithelial cells. J Cell Sci 114: 1331-1341.
3. Lane MC, Alteri CJ, Smith SN, Mobley HLT (2007) Expression of flagella is coincident with uropathogenic *Escherichia coli* ascension to the upper urinary tract. Proc Natl Acad Sci 104: 16669-16674.
4. Datsenko KA, Wanner BL (2000) One-step inactivation of chromosomal genes in *Escherichia coli* K-12 using PCR products. Proc Natl Acad Sci 97: 6640-6645.
5. Uzzau S, Figueroa-Bossi N, Rubino S, Bossi L (2001) Epitope tagging of chromosomal genes in Salmonella. *Proc Natl Acad Sci U S A* 98(26):15264–15269.
