# Supplementary material for: Ethanolamine Signaling Promotes Salmonella Niche Recognition and Adaptation during Infection
Source: PLoS Pathog. 2015 Nov 13;11(11):e1005278. doi: 10.1371/journal.ppat.1005278 (PMC4643982; doi:10.1371/journal.ppat.1005278)
Supplement: S2 Table — (PDF) [file ppat.1005278.s013.pdf]

Table S2. Oligonucleotide primers

Target gene	Purpose	Sequence
eutR	λ-Red	5’-ATCATGAAAAAGACCCGTACAGCGAATTTGCACCATCTTTATCATGAAGCGTGTGTAGGCTGGAGCTGCTTC-3’
		5’-TCAATATCAAGCCCATTGCCGCATCCGTTGATGCAGCGTCAACGACGGTTTTCATATGAATATCCTCCTTAG-3’
eutB	λ-Red	5’-GTGACGGTGAAATCACTCGCATTTCCTTCCTGAGGGAACGACTTATGAAAGTGTAGGCTGGAGCTGCTTC-3’
		5’-CCGCGTCATCAGAAGAACAGTGACGGATCGCCCGCCCGTTTGGTCAGACGCATATGAATATCCTCCTTAG-3’
eutR	*eutR* complement	5’-CTAGAAGCTTTTATTGGCGCTGCTGGCATCAGTACG-3’
		5’-CTAGGGATCCTCAAGCCCATTGCCGCATCCGTTGAT-3’
eutR	qRT-PCR	5’-GGGAGGCACCGAGTTTGAA-3’
		5’-GATAACGACGCCCAGAATGG-3’
strB	qRT-PCR	5’-CTACCAGGCGACCGAAATTG-3’
		5’-GTTCCTCAGATGCGGCATACA-3’
ssrB	qRT-PCR/qPCR	5’-GGGAAGCTATCCTGGCTGAATT-3’
		5’-CGCGCAAAGTAAGCAGTTGA-3’
ssaB (spiC)	qRT-PCR	5’-GGATATCAGGGCCGAAGGTAA-3’
		5’-GCAGGATGCCCATCAATAGTC-3’
sseA	qRT-PCR	5’-CAGAGAGCCCCAAAAATAGCA-3’
		5’-CTTCTCGGCCTCCTGGTTAA-3’
ssaG	qRT-PCR	5’-GCCAGGCCATTAATGACAAAA-3’
		5’-TGCGCTTTAATCATCGATTCTG-3’
sifA	qRT-PCR	5’-GTTGTCTAATGGAACCGATAATATCG-3’
		5’-CTACCCCCTCCCTTCGACAT-3’
eutR	protein expression	5’-CCATGGCTAAAAAGACCCGTACAGCGAATTTG-3’
		5’-CCTGCAGGAGCCCATTGCCGCATCCGTTGATG-3’
amp	Gel shift	5’-GGAATTCGAAAGGGCCTCGTGATACGC-3’
		5’-CGGGATCCGGTGAGCAAAAACAGGAAGG-3’
ssrB	Gel shift	5’-ATAGCAAGATTTTCCAATCACTG-3’
		5’- ATGATGATTTCATGATCGTCTAC -3’
ssrB	qPCR	5’-CATCGCGCTGGAGGAACTAA-3’
		5’- GGCCATTGATGCCAGGTAGA -3’
eutS	qRT-PCR	5’-ACACATCGGTTTTCTCGATAGATTT-3’
		5’-CGCCGACGGTGCCATA-3’
ssrA	qRT-PCR	5’-GCAATGCAAACCATCCAGGG-3’
		5’-ATTTTACCGCGTTCCCGAGT-3’
sipC	qRT-PCR	5’-GACGGGCGATCTGATTATGAA-3’
		5’-GACGCCCCTGCAATACCA-3’
16sRNA	qRT-PCR	5’-CCAGCAGCCGCGGTAAT -3’
		5’-TTTACGCCCAGTAATTCCGATT-3’
eutS	qPCR	5’-GCTCACCGTCTCCACTGAAA-3’
		5’-ATTCTGGAACCTGTTGCGCT-3’
kan	Gel shift	5’-CCGGAATTGCCAGCTGGGGCG-3’
		5’-TCTTGTTCAATCATGCGAAACGATCC-3’
eutR	EutR:FLAG	5’-ACCGTCGTTGACGCTGCATCAACGGATGCGGCAATGGGCTGACTACAAAGACCATGACGG-3’
		5’-CACGTTATCAGCAACCGGAGAGCCTCCCCATCAATATCAACATATGAATATCCTCCTTAG-3’
